# A machine learning approach to integrating genetic and ecological data in tsetse flies (*Glossina pallidipes*) for spatially explicit vector control planning

**DOI:** 10.1111/eva.13237

**Published:** 2021-05-05

**Authors:** Anusha P. Bishop, Giuseppe Amatulli, Chaz Hyseni, Evlyn Pless, Rosemary Bateta, Winnie A. Okeyo, Paul O. Mireji, Sylvance Okoth, Imna Malele, Grace Murilla, Serap Aksoy, Adalgisa Caccone, Norah P. Saarman

**Affiliations:** ^1^ Department of Ecology and Evolutionary Biology Yale University New Haven CT USA; ^2^ Department of Environmental Science, Policy, & Management University of California Berkeley CA USA; ^3^ School of the Environment Yale University New Haven CT USA; ^4^ Department of Ecology and Genetics Uppsala University Uppsala Sweden; ^5^ Department of Anthropology University of California Davis CA USA; ^6^ Biotechnology Research Institute Kenya Agricultural and Livestock Research Organization Kikuyu, Nairobi Kenya; ^7^ Department of Biomedical Sciences and Technology School of Public Health and Community Development Maseno University Maseno, Kisumu Kenya; ^8^ Centre for Geographic Medicine Research Coast Kenya Medical Research Institute Kilifi Kenya; ^9^ Vector and Vector Borne Diseases Research Institute Tanzania Veterinary Laboratory Agency Tanga Tanzania; ^10^ Department of Epidemiology of Microbial Diseases Yale School of Public Health New Haven CT USA; ^11^ Department of Biology Utah State University Logan UT USA

**Keywords:** disease vector, gene flow, habitat suitability, landscape genetics, random forest, spatial modeling

## Abstract

Vector control is an effective strategy for reducing vector‐borne disease transmission, but requires knowledge of vector habitat use and dispersal patterns. Our goal was to improve this knowledge for the tsetse species *Glossina pallidipes*, a vector of human and animal African trypanosomiasis, which are diseases that pose serious health and socioeconomic burdens across sub‐Saharan Africa. We used random forest regression to (i) build and integrate models of *G*. *pallidipes* habitat suitability and genetic connectivity across Kenya and northern Tanzania and (ii) provide novel vector control recommendations. Inputs for the models included field survey records from 349 trap locations, genetic data from 11 microsatellite loci from 659 flies and 29 sampling sites, and remotely sensed environmental data. The suitability and connectivity models explained approximately 80% and 67% of the variance in the occurrence and genetic data and exhibited high accuracy based on cross‐validation. The bivariate map showed that suitability and connectivity vary independently across the landscape and was used to inform our vector control recommendations. Post hoc analyses show spatial variation in the correlations between the most important environmental predictors from our models and each response variable (e.g., suitability and connectivity) as well as heterogeneity in expected future climatic change of these predictors. The bivariate map suggests that vector control is most likely to be successful in the Lake Victoria Basin and supports the previous recommendation that *G*. *pallidipes* from most of eastern Kenya should be managed as a single unit. We further recommend that future monitoring efforts should focus on tracking potential changes in vector presence and dispersal around the Serengeti and the Lake Victoria Basin based on projected local climatic shifts. The strong performance of the spatial models suggests potential for our integrative methodology to be used to understand future impacts of climate change in this and other vector systems.

## 1 INTRODUCTION

Worldwide, vector‐borne diseases account for more than 17% of all infectious diseases in humans and represent a significant socioeconomic burden through decreases in livestock milk production, birth rates, weight gain, and survival (Chanie et al., 2013; Narladkar, 2018; Rohr et al., 2019). The potential of a vector to transmit a pathogen is heterogeneous across the landscape because of variation in the disease, vector, and risk of contact between host and vector. Variation in distribution is caused by complex evolutionary and ecological interactions between the organism and the local environment over multiple generations. Ultimately, variation in vector survival and dispersal are two components that most strongly influence long‐term disease transmission. Both survival and dispersal can be modeled spatially as estimates of habitat suitability and genetic connectivity (Bouyer et al., 2015; Dicko et al., 2014; Hirzel & Lay, 2008), which can improve our ability to plan and implement disease control interventions.

Tsetse flies (genus *Glossina*) are obligate vectors of animal and human African trypanosomiasis (AAT and HAT, respectively). These diseases pose serious socioeconomic and health burdens to sub‐Saharan Africa. In Kenya and Tanzania, HAT and AAT are transmitted most often by tsetse of the species *Glossina pallidipes*. Although there have only been a few cases of HAT reported recently in the study area (Franco et al., 2014; World Health Organization, 2020), both Kenya and Tanzania remain classified by the World Health Organization (WHO) as regions of HAT public health concern because of lack of control and surveillance activities (Franco et al., 2020). In contrast to HAT, AAT is widespread throughout the *G*. *pallidipes* range in Kenya and Tanzania. Previous empirical studies and mathematical modeling have indicated that *G*. *pallidipes* populations could be reduced to levels that minimize AAT transmission through vector control strategies such as bush clearance, ground spraying using insecticides, odor‐baited traps, and insecticide‐impregnated targets (Bourn et al., 2001; Davis et al., 2011; Gilbert et al., 2016; Medlock et al., 2013; Ndeffo‐Mbah et al., 2019; Pandey et al., 2015).

Vector control has been used to mitigate damage done by AAT and HAT in east Africa since the 1960 s (Bourn et al., 2001). However, population rebounds in *G*. *pallidipes* are thought to jeopardize the long‐term success of AAT control in the region (Ilemobade, 2009; Rogers & Randolph, 1985). Insect survival outside of the treated areas and subsequent recolonization of treated areas are thought to contribute to population rebounds (Bourn et al., 2001; Okeyo et al., 2017). Knowledge of the environmental factors associated with *G*. *pallidipes* survival and dispersal can improve our ability to predict where tsetse flies may be able to survive vector control campaigns and potential routes of recolonization. Tsetse flies are known to be sensitive to environmental conditions (Brightwell et al., 1992; Hargrove, 2004; Rogers & Randolph, 1991). Variables such as temperature and precipitation have been shown to affect birth rates, death rates, and development of tsetse flies (Hargrove, 2004), while temperature and humidity are known to affect dispersal distance (Brightwell et al., 1992). Understanding of survival and dispersal enables strategic planning that will reduce the risk of population rebounds and thus vector re‐emergence following control efforts.

Advances in spatial modeling and machine learning approaches have improved predictions of species distributions and dispersal patterns by integrating ecological and genetic data (Bouyer et al., 2015; Dicko et al., 2014; Hether & Hoffman, 2012; Hirzel & Lay, 2008; Manel et al., 2003; Pless et al., 2021). In particular, random forest regression, a widely used machine learning method, allows for modeling of nonlinear relationships across landscapes without overfitting (Liaw & Wiener, 2002; Prasad et al., 2006; Rehfeldt et al., 2006). These advantages enable the use of correlated variables and ecological data that violate parametric assumptions (Breiman, 2001; Garzón et al., 2006; Liaw & Wiener, 2002; Murphy et al., 2010; Wagner & Fortin, 2005), contributing to the feasibility of modeling complex landscape‐level factors, such as habitat suitability and genetic connectivity in vectors (Pless et al., 2021).

**FIGURE 1:**
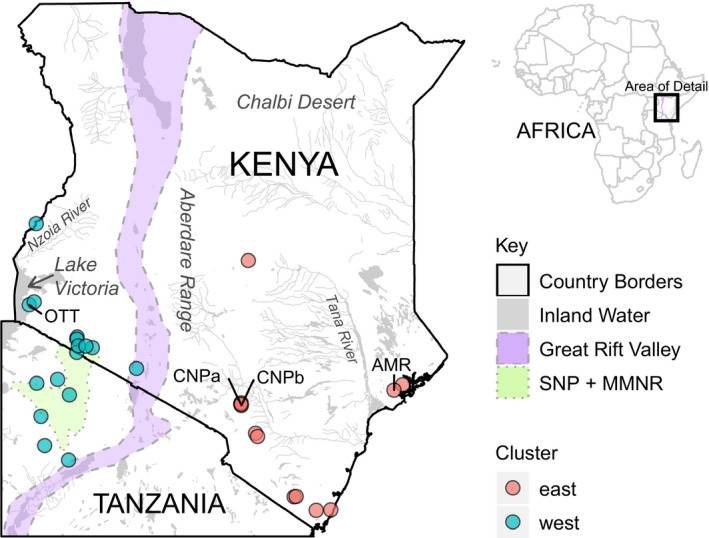
Map of sampling sites in Kenya and Tanzania, color coded by genetic cluster. The boxed area of detail is the location of the study region in Africa. The approximate area of the Serengeti ecosystem is shaded in green (combination of the Maasai Mara National Reserve and the Serengeti National Park), and the approximate outline of the Great Rift Valley is shaded in purple. The three new sampling sites for this study (OTT, CNP, and AMR) are labeled. CNP was split into CNPa and CNPb for our analysis as some trap locations from this sampling site were found to be further than two kilometers apart (see methods). This map was created using the R packages “ggplot2” (Wickham, 2016), “raster” (Hijmans, 2019), and “rgdal” (Bivand et al., 2019) with publicly available data from DIVA‐GIS (March 2020; http://www.diva‐gis.org), Map Library (March 2020; http://www.maplibrary.org), World Map (March 2020; https://worldmap.harvard.edu), and MaMaSe (March 2020; http://maps.mamase.org)

In this paper, we take advantage of such recent methodological developments in spatial modeling to achieve two goals: to (i) build and integrate models of *G*. *pallidipes* habitat suitability and genetic connectivity across Kenya and northern Tanzania (Figure 1) and (ii) provide novel, spatially explicit vector control recommendations. We use field records and microsatellite genotypic data from published data (Bateta et al., 2020; Cecchi, 2002; Okeyo et al., 2017, 2018) with the addition of three new sampling sites. We developed our analysis strategy in collaboration with Pless et al. (2021) to enable both the identification of environmental correlates of vector habitat suitability and genetic connectivity (from here forward referred to simply as suitability and connectivity) and mapping of these predictions across the landscape. Additionally, we integrated outputs with a novel application of bivariate mapping to identify geographic regions with distinct risks and opportunities for *G*. *pallidipes* vector control. Specifically, we provide vector control recommendations that consider predicted risks of population rebounds, corridors of recolonization, and isolated populations likely to be feasibly eradicated locally and/or used in the development of novel control strategies. Although methodology for predicting vector response to climate change, especially in predicting future connectivity, has not been fully developed, our study takes a first step by demonstrating feasibility of using basic environmental predictors available under climate change scenarios to predict suitability and connectivity. We do not extend this to projecting future suitability and connectivity because of challenges with validating predictions under novel conditions, and accounting for complex biological factors such as demography (Dormann, 2007; Urban et al., 2016; Yates et al., 2018). However, we do use climate change projections of the most important predictors in our models to identify geographic areas of high priority for monitoring for changes in tsetse fly presence and movement. Results indicate strong performance of our methodology, highlighting the utility of machine learning for informing current and future vector control across Kenya and Tanzania.

## 2 METHODS

### 2.1 *Glossina pallidipes* biology and distribution in the study area

*Glossina pallidipes* is a member of the *G*. *morsitans* group and is considered a savannah species. The distribution of *G*. *pallidipes* is limited to savannah habitat and extends into Ethiopia in the north, the Democratic Republic of the Congo and Uganda in central Africa, Kenya, and Tanzania in central east Africa, and Mozambique and Zambia in southern east Africa (Ford, 1971; Jordan, 1993; Rogers & Randolph, 1985; Rogers & Robinson, 2004). However, the boundaries of savannah habitat mean that the continuous distribution of *G*. *pallidipes* does not extend into Ethiopia or Uganda, is limited within Kenya to areas south of Mt Kenya, and is limited within Tanzania to the Serengeti ecosystem and a band of habitat along the coast of the Indian Ocean (Cecchi et al., 2008; Ford, 1971; Jordan, 1993; Ngari et al., 2020; Pollock, 1982; Rogers & Randolph, 1985; Rogers & Robinson, 2004). Previous work has shown that for *G*. *pallidipes*, the tsetse fly belts recognized by the Kenya Tsetse and Trypanosomiasis Eradication Council (KENTTEC) are not necessarily ecologically or evolutionarily distinct. Instead, there is a weak genetic break of recent origin with current gene flow between the Lake Victoria Basin and the Serengeti ecosystem and a strong biogeographic break caused by the expansion of the Great Rift Valley in central Kenya (Faith et al., 2016; Lehmann et al., 1999; Linder et al., 2012; Wilfert et al., 2006; Wüster et al., 2007; Figure 1) that separates populations east and west of the valley. Thus, it was suggested by Bateta et al. (2020) that all populations east of the valley should be managed together. With this in mind, the biologically relevant geographic scope for management of *G*. *pallidipes* in Kenya extends from the Lake Victoria Basin at the border of Uganda and Kenya east to the Indian Ocean and south to the edge of the Serengeti ecosystem in Tanzania.

*Glossina pallidipes* has a generation time of approximately five per year, has variable dispersal rates on the order of 0.1–10 km per individual/generation (Brightwell et al., 1992; Cuisance et al., 1985; Hargrove, 1981; Rogers, 1977), and goes through population contractions during several arid periods of the year and expansions during rainy seasons (Camberlin & Wairoto, 1997; Devisser et al., 2010; Nnko et al., 2017; Pollock, 1982; Rogers & Randolph, 1985). These population fluctuations make it difficult to identify the extent of the distribution with trapping efforts, as a negative result does not necessarily mean low density at all times of year. These challenges have prompted extensive efforts by KENTTEC and others to collect across multiple seasons and years for the full distribution of *G*. *pallidipes* in the region (Bateta et al., 2020; Cecchi et al., 2008; Ngari et al., 2020; Okeyo et al., 2017, 2018; Opiro et al., 2017). Nonetheless, copyright of much of the sampling efforts by the Kenyan government makes these data unavailable to the scientific community (Ngari et al., 2020), leaving urgent need for a publicly available up‐to‐date suitability model that is based on environmental conditions and is well integrated with knowledge of tsetse dispersal patterns.

### 2.2 Summary of data inputs

#### 2.2.1 A1. Field survey occurrence data and background points

**FIGURE 2:**
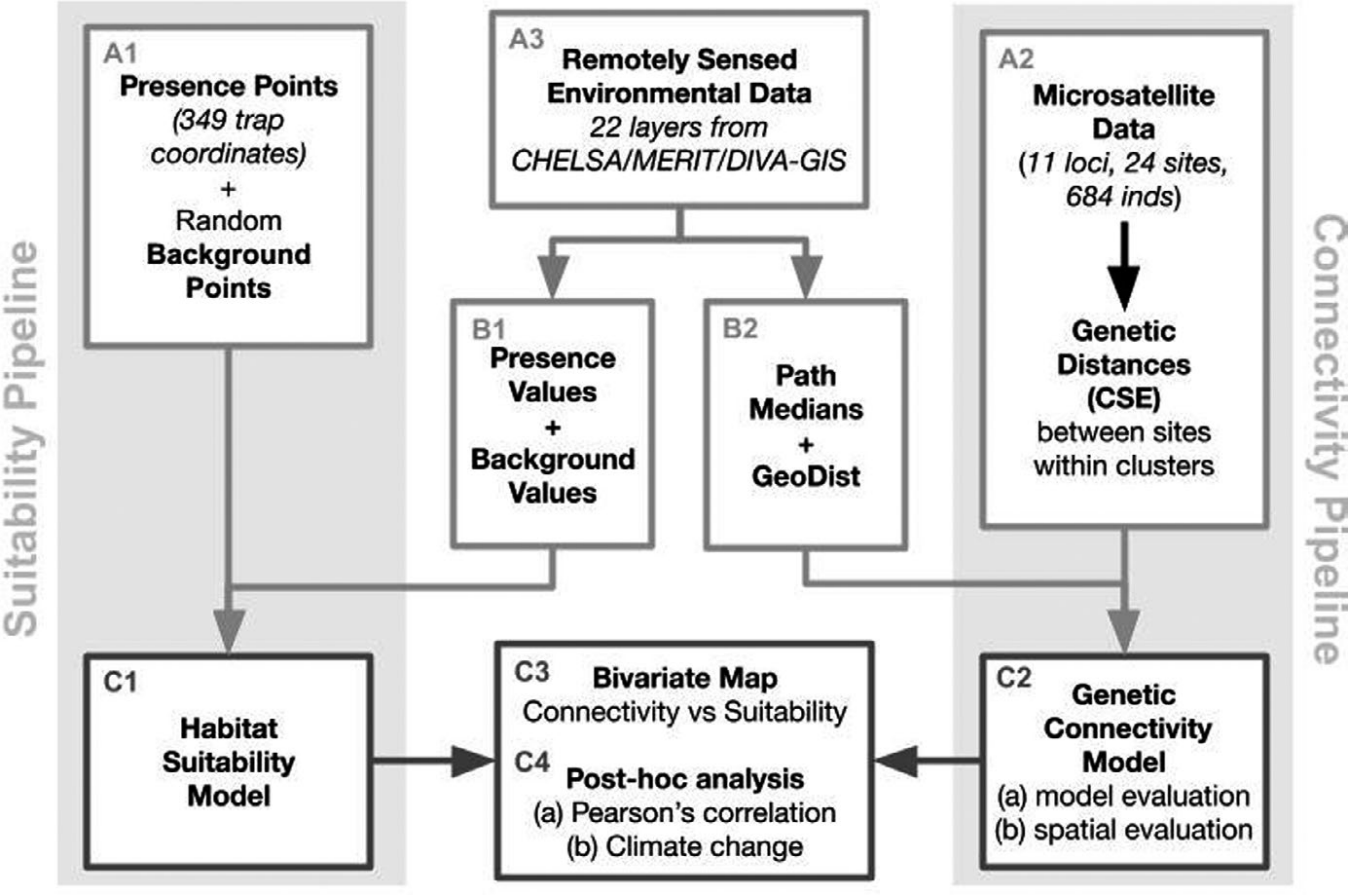
Diagram of simplified methods. Light gray shaded boxes indicate the separate pipelines for the suitability (A1, C1) and connectivity (A2, C2) models. The original data inputs are presence‐background data (A1) and microsatellite data (A2) from flies caught during trapping surveys in Kenya and northern Tanzania as well as remotely sensed data from CHELSA, MERIT, and DIVA‐GIS repositories (A3). See methods for more details on calculation of genetic distances (A2), manipulation of environmental data (B1, B2), and selection of background points (A1). Dark gray outlined boxes (C1, C2, C3, C4) illustrate the final outputs of the pipelines (C1, C2), the bivariate map of connectivity and suitability (C3), and post hoc analyses (C4)

The field data were from trapping surveys carried out from 2015 to 2019 across Kenya and northern Tanzania (Bateta et al., 2020; Okeyo et al., 2017, 2018). Bi‐conical and Ngu traps were placed in the field at sampling sites in clusters of 3–5 traps separated by less than 5 km and were left out for either 24 or 48 h. The sampling used in this study was from a concerted effort by our research group to comprehensively sample the *G*. *pallidipes* distribution in Kenya, as well as the connected habitat across political boundaries (i.e., Tanzania, as the *G*. *pallidipes* distribution does not extend continuously into Uganda; Pollock, 1982). There is also evidence that the sampling effort was comprehensive, as there were an equal number of visited sites with no fly catches as those with fly catches that were within the expected distribution (Bateta et al., 2020). Locations of traps with flies in them were used as presence points in the suitability model (A3, Figure 2), and live flies were preserved in 80% ethanol for microsatellite genotyping. Instead of absence points, we used randomly selected “background” points to characterize the full range of environmental conditions. Background points allow the model to better distinguish the conditions under which species presence is more likely from the overall environmental conditions (Elith et al., 2006; Phillips et al., 2009). Use of background points at a sample size that matches presence points (in this case ~100 once converted to a 1 × 1 km grid raster) has been demonstrated to maximize accuracy in species distribution models (Barbet‐Massin et al., 2012; Elith et al., 2006; Phillips et al., 2009). For background points, we used 10 replicates of 100 randomly sampled points across the geographic scope of our study (longitude of 33.7° to 42.5°, latitude of −4.8° to 5.0°, excluding ocean) using the R package “dismo” (Hijmans et al., 2017).

#### 2.2.2 A2. Microsatellite data

A total of 659 individuals from 29 sampling sites were genotyped at 11 microsatellite loci, with seven to 46 individuals per sampling site. Genetic data collection included 18 sampling sites in Kenya and six sampling sites in northern Tanzania (~15 flies of each sex for each sampling site; A2, Figure 2). Of these, 600 flies from 21 sampling sites were genotyped by Bateta et al. (2020) and Okeyo et al. (2017, 2018). We added 84 flies from three new sampling sites (Figure 1) and genotyped them at the same 11 loci following the protocol described by Okeyo et al. (2017, 2018). Sampling sites containing traps more than two kilometers apart were split such that all traps within sampling sites are less than two kilometers from each other. We calculated pairwise Cavalli‐Sforza and Edwards’ chord (CSE) genetic distance between sampling sites (A2, Figure S3; Cavalli‐Sforza & Edwards, 1967). CSE genetic distance has been shown to perform better than other genetic distance measures when there are missing data and when the relative distances between population pairs are being measured (Bouyer et al., 2015; Pless et al., 2021). To retain only the genetic distances that reflect contemporary environmental conditions rather than more ancient divergences such as those associated with the expansion of the Great Rift Valley (Faith et al., 2016; Lehmann et al., 1999; Linder et al., 2012; Wilfert et al., 2006; Wüster et al., 2007), we only included genetic distances between sampling sites within the two major genetic clusters east and west of the Great Rift Valley that were identified in previous studies (Bateta et al., 2020; Okeyo et al., 2018) and confirmed here with DAPC (File S1; Jombart, 2008).

#### 2.2.3 A3. Remotely sensed environmental data

Predictor variables for both the suitability and connectivity models were based on 1‐kilometer resolution environmental raster layers of 19 bioclimatic variables, slope, altitude, and river density (A3, Figure 2). Although including more predictor variables (e.g., host availability, landcover) may have potential to improve the model, we chose to limit our selection to variables that are either unchanging on relevant timescales of decades and centuries (i.e., slope, altitude, and river location) or publicly available as forecasts under four different emissions scenarios based on 36 different multiple climate change scenarios (Karger et al., 2017, i.e., 19 climatic variables reflecting temperature and precipitation, i.e., temperature‐ and precipitation‐based climatic variables) allow us to visualize predicted change in climate variables important in our models.

The 19 bioclimatic variables were temperature and precipitation based (Table S1) and were calculated from raster files downloaded from Climatologies at High Resolution for the Earth's Land Surface Areas (CHELSA; Karger et al., 2017) for the time span of 2008–2013 with the R package “dismo” (Hijmans et al., 2017). We used seasonal bioclimatic variables based on the precipitation seasonality trends observed in the study area, rather than the default quarterly estimates, to more accurately capture the seasonal variation relevant to the ecology of the region (Table S1; Figure S1). Slope and altitude raster files were downloaded from Geomorpho90m dataset (Amatulli et al., 2020) and Multi‐Error‐Removed Improved‐Terrain (Yamazaki et al., 2017), respectively. Following methods described in Pless et al. (2021), we created a river density layer in the R package “KernSmooth” (Wand, 2015) based on river shapefiles downloaded from DIVA‐GIS (March 2020; http://www.diva‐gis.org). The final raster layers were clipped to the extent of Kenya and northern Tanzania (longitude of 33.7° to 42.5°, latitude of −4.8° to 5.0°) and projected to the WGS‐84 coordinate reference system in the R package “rgdal” (Bivand et al., 2019).

All spatial data, including the environmental inputs and results from the models (see below), were visualized using the R packages “raster” (Hijmans, 2019), “rgdal” (Bivand et al., 2019), “rgeos” (Bivand & Rundel, 2020), and “ggplot2” (Wickham, 2016), and figures were produced using R packages “ggpubr” (Kassambara, 2019), “gridExtra” (Auguie, 2017), “patchwork” (Pedersen, 2020), and “ggrepel” (Slowikowski, 2020).

### 2.3 Random forest model of habitat suitability

#### 2.3.1 B1. Environmental point values

For the suitability model, we used environmental values extracted at the coordinates of the presence (*n* = 349 trap locations) and background points (*n* = 100 per model replicate) for the 22 environmental variables using the R package “raster” (Hijmans, 2019).

#### 2.3.2 C1. Building and projecting the RF model

Following methods described in Hill et al. (2017), we built, evaluated, and projected our suitability model with the R packages “biomod2” (Thuiller et al., 2019), “raster” (Hijmans, 2019), “sp” (Pebesma & Bivand, 2005), and “rgdal” (Bivand et al., 2019) using presence/background scored as 1/0, respectively, as the response variable and 22 environmental values extracted at these coordinates as the explanatory variables (B3, A3, B4, Figure 2). We treat the binary (1/0) data as a continuous response variable (i.e., ran a regression model) in order to end up with a continuous measure of suitability. Hence, we assessed model performance with the *R*‐squared generated internally by the random forest algorithm, which is based on a bootstrapping procedure that repeatedly selects a random sample (with replacement) of training sets and compares the average predictions with the testing sets that were left out of the model (Breiman, 2001; Liaw & Wiener, 2002). We evaluated variable importance using increase in node purity, which is calculated by taking the decrease in the residual sum of squares (RSS) as the result of splitting on each variable and averaging it across all trees (Liaw & Wiener, 2002). We choose to evaluate variable importance in this way rather than using percent increase in mean square error from permuting each variable (another evaluation option provided by random forest) because increase in node purity is not sensitive to correlation between variables. To evaluate model performance, we used a 10‐fold cross‐validation procedure and calculated the true skill statistic (TSS) and the area under the receiver operating curve (AUC) (Allouche et al., 2006).

### 2.4 Combining suitability output with previous models

The existing suitability map available for *G*. *pallidipes* in eastern Africa (Cecchi, 2002; Cecchi et al., 2008) needed to be updated because it was based on trapping records that were more than 15 years old and had obvious inaccuracies. The most notable inaccuracy is the prediction of low suitability in the Serengeti ecosystem, a region known to harbor *G*. *pallidipes* and that had high capture rates in trapping records used in this study. However, the raw data are property of the Government of Kenya (Kenya Tsetse and Trypanosomiasis Eradication Council), and we have not been granted access (Cecchi, 2002; Cecchi et al., 2008; Ngari et al., 2020). Thus, instead of building a comprehensive model, as would have been our preference, we combined our map with the existing map. We combined the maps by taking the maximum predicted suitability for each pixel from the two maps, the most conservative way possible given that for vector control, it is better to overpredict than under‐predict vector presence.

### 2.5 Random forest model of genetic connectivity

#### 2.5.1 B2. Environmental path data and geographic distance

We extracted the median value along straight paths (*n* = 198 paths) between sampling sites (*n* = 29 sampling sites) within genetic clusters for each of the 22 environmental variables (B3, Figure 2) using the R package “raster” (Hijmans, 2019). We chose to use the median value as opposed to the mean because it is not as affected by the presence of outliers. We included two additional explanatory variables, (i) mean kernel density of sampling effort and (ii) geographic distance to ensure our model accounted for spatial auto‐correlation (File S1; Shi et al., 2019; Souris & Demoraes, 2019). We created a sampling density layer in the R package “KernSmooth” (Wand, 2015; File S1) and estimated the median value along the 198 straight paths, as was done for the 22 environmental variables. Geographic distance was estimated following Bouyer et al. (2015) by creating a uniform raster (all 1 × 1 km pixels were assigned a value of 1) and summing values along the 198 straight paths.

The inclusion of these variables was necessary because spatial auto‐correlation is an almost ubiquitous confounding factor in landscape‐level studies. Auto‐correlation is especially pronounced in population genetic studies because genetic distance is expected to be correlated with geographic distance under neutral conditions (Rousset, 1997; Wright, 1943). This was of particular concern in this study because genetic and geographic distance were reported to be correlated in some subsets of this dataset (Bateta et al., 2020), a result we confirmed with Mantel tests (File S1; Dray & Dufour, 2007; Mantel, 1967). Nonetheless, we think that the spatial modeling approach implemented is appropriate because we were able to demonstrate with Anderson‐Darling *k*‐means tests (Scholz & Zhu, 2019) that the majority of variation in genetic distance remained unexplained in models that considered geographic distance alone (File S1).

#### 2.5.2 C2. Building and projecting the connectivity model

Our connectivity model was built with the full dataset (29 sampling sites, 198 Paths) using the packages “randomForest” (Liaw & Wiener, 2002), “raster” (Hijmans, 2019), “spatstat” (Baddeley & Turner, 2005), and “sp” (Pebesma & Bivand, 2005). We built a random forest model using CSE genetic distance between sampling site pairs as a proxy for connectivity (B3, C2, Figure 2). This model was projected across Kenya and Northern Tanzania to create a map of predicted connectivity using the environmental data and sample density rasters, as well as the raster with uniform values of 1 used to estimate geographic distance following Bouyer et al. (2015). This procedure essentially assigned the geographic distance between each pixel and itself to 1 km in the projections of the model. As in the suitability model, we assessed model performance with the internally generated *R*‐squared and variable importance using increase in node purity.

#### 2.5.3 C2a. Model evaluation

To allow for evaluation of the connectivity model's performance in different subsets of the data, we used leave‐one‐out cross‐validation. For each run of the cross‐validation, the root mean square error (RMSE) was calculated based on testing data not included in the training of the model. We assessed the accuracy of our models by generating a null distribution of 100 RMSE values (i.e., values expected by chance for this type of modeling) from models trained on randomly shuffled data and used this as a benchmark against which to compare our observed RMSE distribution using Welch's *t* tests (File S1).

#### 2.5.4 C2b. Spatial evaluation

We estimated the accuracy of the projections for each run of the leave‐one‐out cross‐validation by extracting the median CSE genetic distance along straight paths between sampling sites from the testing data. Comparing these spatially predicted CSE values to the observed CSE values allowed us to estimate RMSE values that reflected the accuracy of the projected connectivity map. As we did for the model evaluation, we compared the observed spatial RMSE values to null distributions generated with shuffled data (see paragraph above, File S1).

### 2.6 Integrating and interpreting outputs to inform vector control

#### 2.6.1 C3. Integrating habitat suitability and genetic connectivity models

We created a bivariate map of predicted suitability and connectivity (C3, Figure 2; File S2) using R packages “raster” (Hijmans, 2019), “rgdal” (Bivand et al., 2019), “classInt” (Bivand, 2018), and “XML” (Lang et al., 2019). We masked all probability of presence values less than ten percent in the suitability model projection such that comparisons were not made where tsetse flies were expected to be absent. More information about the creation of this bivariate map can be found in File S1 and File S2.

#### 2.6.2 C4a. Post hoc visualization of local correlations

The random forest approach we use in this study has several advantages over other standard modeling approaches, such as simple linear regression, including greater flexibility and higher predictive power when modeling complex, nonlinear relationships (File S1). However, as is the case with many machine learning methods, the trade‐off for this superior performance is more complexity and less interpretability. Thus, to gain a better understanding of the environmental drivers of suitability and connectivity, we used the corLocal() function in the R package “raster” (Hijmans, 2019) to calculate the Pearson's correlation coefficient between projections of the response variables of interest (i.e., suitability or connectivity (1‐scaled genetic distance)) and the top predictor variables identified by our random forest models.

#### 2.6.3 C4b. Post hoc visualization of predicted environmental change

Global warming is expected to affect tsetse fly distribution and connectivity (Bourn et al., 2001), making knowledge of the environmental drivers of tsetse fly distribution and connectivity under current and future conditions a valuable part of planning vector control strategy. For short‐term planning, the bivariate maps we built can provide specific vector control recommendations for different categories of landscape in Kenya and northern Tanzania (see above). Long‐term planning is more difficult and is influenced by more uncertainties. Although it would be ideal to project our models under future conditions, the methodology for this is not fully developed. There are outstanding challenges in transferring models to novel conditions, such as accounting for the effects of biological mechanisms (i.e., demography, species interactions, and evolutionary change), quantifying uncertainty, and assessing transferability (Dormann, 2007; Urban et al., 2016; Yates et al., 2018). Instead, we take an alternative approach that avoids unrealistic assumptions about the effects of biological mechanisms as well as problems with model validation and transferability: We provide estimates of predicted change in the most important environmental variables from our models of *G*. *pallidipes* suitability and connectivity. In this way, our approach informs which geographic regions will experience environmental change that may affect *G*. *pallidipes* vectoring capacity, and we interpret these as the regions that should be monitored for changes in vector presence and dispersal. Even though we cannot presently define the magnitude or direction of future changes in connectivity and suitability given the limitations of our data and models, knowing where to expect relevant environmental change could be used to optimize future monitoring efforts. We estimated the predicted change of the most important environmental variables from the suitability and connectivity models under the NASA RCP 4.5 climate change model for 2041–2060, calculated by subtracting the present environmental layer (an average across 2008–2013) from the future environmental layer. Both present and future environmental layers for each variable were sourced from CHELSA (Karger et al., 2017).

## 3 RESULTS

### 3.1 Habitat suitability model

#### 3.1.1 Full model results

The mean *R*‐squared for the 10 suitability models built using all presence points and each of the 10 sets of background points was 0.80 (SD = 0.02), indicating that on average 80% of the variance in suitability was explained by the predictor variables. The most important variable for six of the 10 models, based on the increase in node purity, was the maximum temperature of the warmest month (Figure 5a, Figure S6A), and for the remaining four models, the most important variable was the temperature annual range (Figure 5a, Figure S6A). These variables suggest that temperature was the most predictive climatic variable of *G*. *pallidipes* presence in tsetse fly traps.

#### 3.1.2 Model evaluation

The random forest suitability models demonstrated high accuracy across all 10 folds of the cross‐validation and all 10 sets of randomly selected background points. The mean AUC of all sets and folds was 0.99 (SD = 0.01) and the AUC never fell below 0.92, indicating an overall favorable ratio between sensitivity (low false negatives) and specificity (low false positives) across all thresholds. The mean true skill statistic (TSS) of all sets and folds was 0.96 (SD = 0.02) and the TSS never fell below 0.80, indicating that the models were both sensitive and specific when discerning presence and absence points based on the threshold that optimizes the TSS as determined in “biomod2” (Thuiller et al., 2019).

### 3.2 Genetic connectivity model

#### 3.2.1 Full model results

The full model of connectivity (Figure S5B) performed well with a *R*‐squared of 0.67, indicating that on average 67% of the variance in genetic distance was explained by the predictor variables. Results from the increase in node purity analysis indicated that precipitation of the driest season was the most important variable in the final model of connectivity (Figure 5b, Figure S6B). Increase in node purity measures how well the variable of interest can be used to split the data, suggesting that precipitation may be an important environmental driver of tsetse fly movement and/or survival and reproduction after relocating.

#### 3.2.2 Model evaluation

**FIGURE 3:**
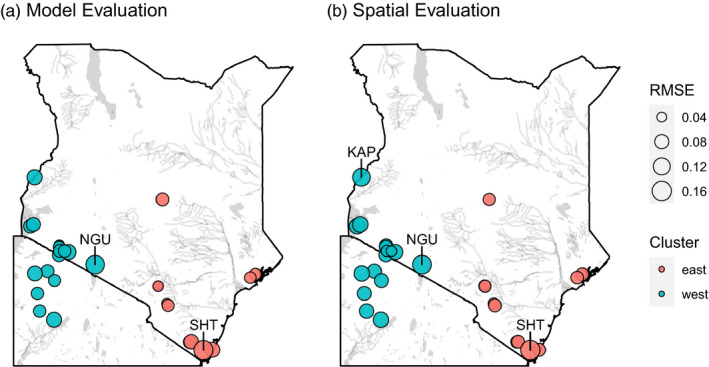
Maps of RMSE values for each sampling site from the leave‐one‐out cross‐validation results. Sampling sites are color coded by genetic cluster: (a) RMSE values from external validation of the genetic connectivity model and (b) RMSE values from the spatial evaluation of the genetic connectivity map (the projection of the genetic connectivity model). Sites with high error compared to other sites and to the null models are labeled (File S1)

The mean RMSE from the leave‐one‐out cross‐validation was 0.07 (SD = 0.03) across all 29 runs (all 29 sampling sites; Figure 3a). The mean RMSE for testing sampling sites from the east was 0.06 (SD = 0.03) and from the west was 0.08 (SD = 0.02), and this difference was not significant (*t*(20.799) = −1.18, *p* = 0.25). Based on *t* tests, the RMSE values from our model were significantly lower (*p*‐value <0.05) than the RMSE values from the null models (mean = 0.11, SD = 0.02; File S1).

#### 3.2.3 Spatial evaluation

Spatial evaluations were calculated by comparing the median genetic distances from straight paths between sampling sites along the projected model surface to the observed genetic distances between sampling sites. The mean RMSE from the spatial evaluation of the model projections was 0.08 (SD = 0.03) across all 29 leave‐one‐out cross‐validation runs (Figure 3b). The mean spatial RMSE for testing sampling sites from the east was 0.07 (SD = 0.03) and from the west was 0.09 (SD = 0.03), but this difference was not significant (*t*(24.261) = −2.04, *p* = 0.05). Based on *t* tests, the spatial RMSE values from our model were significantly lower (*p*‐value <0.05) than the spatial RMSE values from the null models (mean = 0.11, SD = 0.02; File S1).

### 3.3 Integrating and interpreting outputs to inform vector control

#### 3.3.1 Integrating habitat suitability and genetic connectivity

**FIGURE 4:**
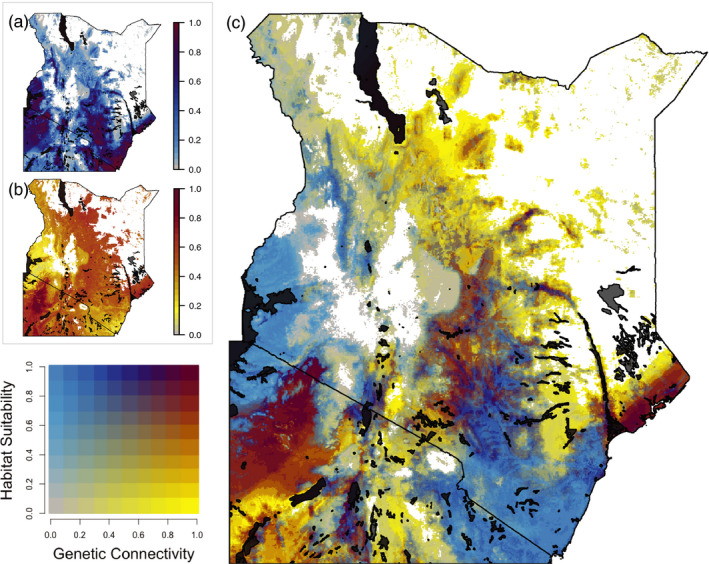
Predicted genetic connectivity and habitat suitability based on machine learning (random forest) models. White areas in all three maps are regions where the predicted probability of *G*. *pallidipes* presence is less than ten percent, based on the habitat suitability map. (a) Scaled map of habitat suitability (combination of our final model and the FAO model), (b) scaled and transformed (1‐scaled genetic distance) map of genetic connectivity, and (c) bivariate map of genetic connectivity versus habitat suitability. The bivariate legend in the bottom left‐hand corner shows the corresponding colors for the different percentiles of genetic connectivity and habitat suitability (dark red: high genetic connectivity/high habitat suitability, yellow: high genetic connectivity/low habitat suitability, blue: low genetic connectivity/high habitat suitability, gray: low genetic connectivity/low habitat suitability)

The bivariate map of the final suitability and connectivity models showed heterogeneous spatial patterns in suitability and connectivity (Figure 4). Low suitability was predicted primarily in the Chalbi Desert (Figure 1) and around the center of the Great Rift Valley in Kenya (Figure 4a). Regions of high connectivity and high suitability included the northeastern part of Tanzania (around the Serengeti area), central Kenya (along the Aberdare Mountain Range, Figure 1), and a small section of the eastern coast of Kenya (Figure 4c). In Kenya, the southern tip (extending into Tanzania) and the area to the west of the Great Rift Valley (around Lake Victoria, Figure 1) had high predicted suitability, but low connectivity (Figure 4c).

#### 3.3.2 Post hoc visualization of local correlations

**FIGURE 5:**
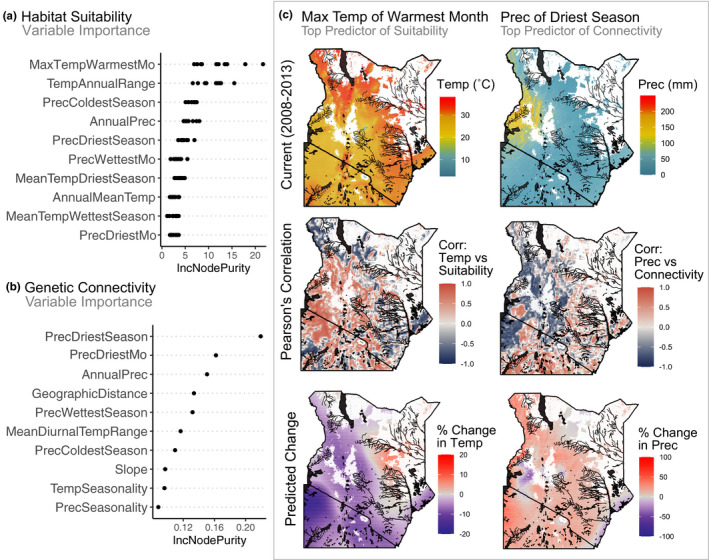
Variable importance plots for (a) the 10 replicate habitat suitability models and (b) the final genetic connectivity model. Only the top 10 most important variables are shown, for the full variable importance plots see Figure S6. The R package “randomForest” measures importance based on the increase in node purity (IncNodePurity). Variables correspond to those described in Table S1. (c) Post hoc analyses of the most important predictor variable for habitat suitability (left column) and genetic connectivity (right column). The first row of maps shows the current environmental conditions (color palette from the “wesanderson” package; Ram & Wickham, 2018). The second row of maps shows the local Pearson's correlations between the top predictor variables and response variables of interest (i.e., maximum temperature of the warmest month vs suitability (probability of presence) and precipitation of the dries season vs connectivity (1‐scaled genetic distance). The local correlation coefficients were calculated with the corLocal() function from the R package “raster” (neighborhood size = 21; Hijmans, 2019). The third row shows maps of the predicted future change in the top predictor variables under the NASA RCP 4.5 climate change model for 2041–2060. White areas in all maps are regions where the predicted probability of *G. pallidipes* presence is less than ten percent, based on the habitat suitability model. Abbreviations: Precipitation (Prec), Temperature (Temp), Maximum (Max), Correlation (Corr), Month (Mo)

The maps of Pearson's correlations between the most important predictor variables and the response variables (i.e., suitability and connectivity, respectively) showed spatial variation in the direction and magnitude of associations (Figure 5c). The correlation between maximum temperature of the warmest month (i.e., the most important variable from the suitability model) and suitability was generally positive in the eastern part of Kenya, around the Lake Victoria Basin and following the Great Rift Valley into Tanzania (Figure 5c). In the western part of Kenya, the spatial pattern of correlation was much more patchy, with interspersed areas of positive and negative associations (Figure 5c). The map of correlation between precipitation of the driest season (i.e., the most important variable from the connectivity model) and connectivity had positive patches in eastern Kenya, primarily along rivers, as well as around the Serengeti (Figure 5c). Precipitation of the driest season had a strong, negative correlation with connectivity around the Lake Victoria Basin in western Kenya (Figure 5c).

#### 3.3.3 Post hoc visualization of predicted environmental change

To inform understanding of the impact of climate change on *G*. *pallidipes* connectivity and suitability, we estimated the predicted change over the next 20–40 years (NASA RCP 4.5 climate change model for 2041–2060) of the most important variables from our models (Figure 5, Figure S6). Predicted change in the maximum temperature of the warmest month, the most important variable from the suitability model, indicated that changes in temperature are expected across most of Kenya, with a general increase in temperature in the north and a decrease in temperature in the south and coastal habitats (Figure 5c). Precipitation of the driest season, the most important variable from the connectivity model, is predicted to change fairly homogeneously across the landscape (Figure 5c). A notable deviation from this uniform change is a concentrated patch of predicted decreased precipitation along the eastern shore of Lake Victoria (southwest corner of Kenya; Figure 5c).

## 4 DISCUSSION

The goals of this paper were to (i) build and integrate models of *G*. *pallidipes* suitability and connectivity and (ii) provide spatially explicit vector control recommendations. Both our models demonstrated strong performance and were able to explain a large portion of the variance in suitability and connectivity. Bivariate maps of suitability and connectivity provide evidence that these factors vary independently across the landscape and indicate that the Serengeti comprises an area of high suitability and high connectivity while both the Lake Victoria Basin and southeastern Kenya constitute areas of high suitability and low connectivity. These results suggest that vector control campaigns are likely to be less successful in the Serengeti and more successful in the Lake Victoria basin and southeastern Kenya. We further recommend that future monitoring efforts should focus on tracking potential changes in vector presence and dispersal around the Serengeti and the Lake Victoria Basin based on projected local climatic shifts.

### 4.1 Habitat suitability model

We were able to explain approximately 80% of the variance in suitability with our suitability model, which also demonstrated strong performance based on the 10‐fold cross‐validation for each of the 10 background point replicates. The standard evaluation statistics were close to the best score possible of one (AUC = 0.99 and TSS = 0.96), indicating that the models accurately predicted the testing data during cross‐validation. The suitability model predicted a patchy distribution of habitat concentrated in the southeast of Kenya and around the Lake Victoria Basin. There is a possibility that the model was overfit to our sampling locations, so to be as conservative as possible we combined our final suitability model with the existing FAO model (Cecchi, 2002). The existing FAO model was built from data collected before 2002, making it out of date, and also shows indications of overfitting since there was a gap in sampling that coincided with low predicted suitability in the Serengeti ecosystem despite this region being known to harbor tsetse flies (Cecchi, 2002; Lord et al., 2018). Although the best solution to this problem would have been to include all known presence points from both data sources in this study, this was not possible because of copyright restrictions (Cecchi, 2002; Ngari et al., 2020), so we combined the models to err on the side of overpredicting vector presence.

The most important variable based on increase in node purity, a random forest variable importance measure, was maximum temperature of the warmest month (Figure 5a, Figure S6A). Based on the map of local correlations, maximum temperature of the warmest season generally had a positive effect on suitability across Kenya and Tanzania (Figure 5c). Temperature is known to affect tsetse fly birth rates, mortality, and development (Brightwell et al., 1992; Hargrove, 2004), suggesting that thermal tolerance may be an important driver of *G*. *pallidipes* habitat use.

### 4.2 Genetic connectivity model

The final random forest model of connectivity explained 67% of the variance in genetic distance and performed well based on both direct evaluation of the model predictions and spatial evaluation of the projected map (Figure 3). There were no notable differences in model performance between the two genetic clusters. Two sampling sites (SHT in the east and NGU in the west) had substantially high error values in comparison to the other sites and the null values (Figure 3; File S1). The site in the east (SHT) was an outlier in the genetic distance distribution from the east. These differences are likely the result of the smaller sampling size for this sampling site (*n* = 7) compared to the average sampling size of 23 individuals. The site in the west (NGU) may have low accuracy because its assignment to the eastern genetic lineage was not fully supported in all analyses (Bateta et al., 2020), implying that genetic divergence from current landscape features could have been masked by the stronger signal of divergence from past vicariance events (i.e., expansion of the Great Rift Valley ~2–5 mya; Faith et al., 2016; Lehmann et al., 1999; Linder et al., 2012; Wilfert et al., 2006; Wüster et al., 2007).

The most important variable for the connectivity model was precipitation of the driest season (Figure 5b, Figure S6B). While it is not possible to discern direct causal relationships between environmental variables and connectivity using this methodology, the importance of precipitation of the driest season may be related to the sensitivity of tsetse fly immature life stages to desiccation (Hargrove, 2004). The risk of desiccation in immature stages may limit successful offspring survival until reproduction in migrants. If true, this suggests that migration often occurs over several generations along corridors of high connectivity. This suggestion has been made to explain the much longer migration distances retrieved in genetic studies that consider several generations than migration distances found in ecological field studies that track a single individual (Bateta et al., 2020; Okeyo et al., 2018; Opiro et al., 2017).

The local correlations between precipitation of the driest season and connectivity exhibit variation spatially (Figure 5c). In the west, connectivity generally has a negative association with precipitation during the driest season, especially around the Lake Victoria Basin and parts of the Great Rift Valley (Figure 5c). One possible explanation for this negative association is that flies have to migrate further to find water in regions where there is low precipitation during the dry season; however, it is not possible to distinguish causality using these models.

In eastern Kenya and parts of Tanzania, there are several discontinuous regions, primarily along rivers and part of the Great Rift Valley, where higher connectivity is associated with higher precipitation during the driest season. This difference in the direction of the correlation between connectivity and precipitation suggests that the ecological mechanisms affecting connectivity may vary across Kenya and Tanzania. Adaptive differences between populations could also play a role in establishing different associations between connectivity and climatic variables, something that could be explored in the future using landscape genomics methods to identify adaptive variation in *G*. *pallidipes* associated with climatic variables such as temperature and precipitation. Although valuable, this is outside of the goals of this paper since the microsatellites used target neutral genetic variation.

### 4.3 Integrating habitat suitability and genetic connectivity models

The bivariate map indicates that suitability and connectivity (Figure 4) are not strongly correlated with each other. A large fraction of the study area with high predicted suitability has low predicted connectivity (blue, Figure 4), contradicting the expectation from landscape ecology that suitability facilitates connectivity (Zeller et al., 2012). This may be due to the limitations of the habitat suitability model, which only takes into account abiotic factors (e.g., ignores ecological interactions) and may overpredict suitability (Broennimann et al., 2012; De Araújo et al., 2014). However, it is also possible that the pattern we observe reflects the biological reality that suitability does not always facilitate connectivity in this system and that different ecological constraints are responsible for shaping habitat use and connectivity in *G*. *pallidipes*. For example, habitat use may be more strongly influenced by the risk of thermal stress while migration over multiple generations that results in gene flow may be more strongly influenced by the risk of desiccation in juveniles.

Regardless of the mechanisms controlling heterogeneity in suitability and connectivity, the bivariate map can be used to identify three categories of landscape that will likely require different vector control strategies: areas of (a) high connectivity and high suitability (red, Figure 4), (b) high connectivity and low suitability (yellow, Figure 4), and (c) low connectivity and high suitability (blue, Figure 4).

Areas of (a) high connectivity and high suitability are found primarily in patches centered in the Serengeti ecosystem and central Kenya (Figures 1 and 4). Our models suggest that these regions could support healthy tsetse populations with high dispersal. High recolonization potential within these regions could render internal control efforts ineffective. Instead, it may be more effective to focus on isolating these areas from neighboring habitat by establishing vector control along their perimeters.

Areas of (b) high connectivity and low suitability are found at the margins of the *G*. *pallidipes* distribution (Figure 4). Our models suggest that these regions support high dispersal and could facilitate reinvasion and seasonal migration. Although these areas may not support year‐round tsetse populations that require targeted treatment, they could act as dispersal corridors. Knowledge of these dispersal corridors can help identify areas with low risk of reinvasion when planning spatially explicit eradication efforts and can also inform placement of treatment technology to block dispersal from outside areas.

Areas of (c) low connectivity and high suitability are found in two large patches, one in western Kenya in the Lake Victoria Basin (Figure 4) and another in southeastern Kenya (Figure 4). Our models suggest that these regions could support large tsetse populations, but that there is low connectivity so these populations are therefore likely to be isolated. The presence of isolated populations in these regions could present an opportunity for testing of novel vector control methods as well as local eradication of tsetse flies. The identification of isolated tsetse fly populations using suitability modeling and population genetics has been previously used to plan successful vector control efforts in Senegal that lead to the local eradication of tsetse flies opening new areas for agriculture (Dicko et al., 2014; Solano et al., 2010).

### 4.4 Applications to vector control

Results from the bivariate map can be used to provide regionally specific recommendations for vector control. In the west, there is a noticeable divide between the region of high suitability and low connectivity in the Lake Victoria Basin (Figure 4) and the region of high suitability and high connectivity within the Serengeti ecosystem. This suggests an effective vector control strategy could be a “rolling carpet” approach, moving from the western part of Kenya toward the Serengeti to minimize re‐invasions. This approach is supported by previous findings of high abundance of *G. pallidipes* within the Serengeti National Park and evidence that there are adjacent regions (in farming areas) where vegetation may still be sufficient to support tsetse populations (Lord et al., 2018). Vector control in the west is particularly important because this region includes a tsetse belt that has been found to have high rates of AAT infection in cattle in addition to a significantly high prevalence of AAT‐related disability in human populations (Grady et al., 2011). In the east, a large area of low connectivity and high suitability overlaps with three KENTTEC identified tsetse belts (the Mbeere‐Meru fly belt, the Central Kenya fly belt, and the Coastal fly belt). Bateta et al. (2020) argued that the eastern belts should be treated as one *G*. *pallidipes* population based on the results of their population genetic analysis. Our modeling approach detected continuous highly suitable habitat with no notable breaks in connectivity in these eastern belts, thus generally supporting the conclusion of Bateta et al. (2020) that the eastern belts should be managed as a single unit.

Results from our post hoc analysis can also be applied to future vector control planning. Post hoc analysis from the suitability model indicates that the top predictor, temperature of the warmest month, is projected to change the most in north central Kenya (north of the Tana River) and northern Tanzania in the Serengeti region (Figure 5c). We suggest that these regions should be monitored for changes in tsetse fly presence and abundance (Figure 4) to provide early warning if there are increases in tsetse fly abundance that could extend the region impacted by AAT. For example, a useful experimental approach could be to set up traps along the perimeters of these regions (e.g., along the Serengeti National Park boundaries in Tanzania and range limits north of the Tana River in Kenya) and monitor annually for changes in tsetse fly density approximated by the number of flies caught in traps using a standard trapping protocol (e.g., those of Bateta et al., 2020; Okeyo et al., 2017, 2018).

Post hoc analysis from the connectivity model indicates that the top predictor, precipitation of the driest season, is expected to change uniformly across Kenya (Figure 5c). An exception occurs in a discrete patch along the eastern shore of Lake Victoria (southwest corner of Kenya) which is expected to experience a substantial decrease in precipitation (Figure 5c). We recommend that future studies are designed to detect changes in connectivity across this patch to provide early warning of increased risk of HAT spreading from the Uganda/Kenya border where the most recent HAT cases were detected (World Health Organization, 2020). Alternatively, a decrease in connectivity over time could present an opportunity to efficiently fortify the barrier to HAT spread eastward with minimal vector control effort. A useful experimental setup in this case would be to place traps throughout the region bounded by the Nzoia River, the eastern shore of Lake Victoria, and the Great Rift Valley (east of the Uganda/Kenya border), an area which has not been well sampled in this or previous studies (Figure 1; Bateta et al., 2020; Okeyo et al., 2017, 2018; Ouma et al., 2006). Time series samples should be collected from the same georeferenced localities every 5 years to monitor for changes in dispersal patterns, approximated by changes in genetic distance and population structure. Previous studies have documented temporal genetic differentiation in *G*. *pallidipes* in eastern Africa at this time scale (Okeyo et al., 2017).

Finally, although we did not directly forecast suitability and connectivity in this study, our results represent a first step toward this goal. Our models, built using only environmental predictors that are available for 36 different climate change models under four different emission scenarios (Karger et al., 2017), or are expected to remain constant in the future (e.g., slope and altitude), performed very well, suggesting that these variables can, at least in theory, provide enough environmental information to allow for projections of both suitability and connectivity models under climate change. However, we refrain from projecting our models in this study due to our current inability to validate projections through time and perform adequate sensitivity analyses to explore how robust our predictions would be to uncertainty in the climate projections. As new data and methods become available, we plan to build on these results and use future projections to evaluate climate change risks impacting the spread of AAT and HAT by tsetse flies.

## 5 CONCLUSION

We identified regions that may host resilient tsetse fly populations, potential routes of recolonization, and candidate isolated locations for local eradication and/or development of novel vector control strategies. Our findings suggest that our machine learning approach can accurately predict tsetse habitat use and connectivity and has great potential to improve understanding of animal habitat use and movement in a changing climate. In this study, our choice of environmental variables that are available as future projections is a first step toward making climate change projections. In this study, we did not make future projections of suitability and connectivity because of the unresolved challenges of transferring models to novel future climatic conditions (Dormann, 2007; Urban et al., 2016; Yates et al., 2018). Future studies should work toward developing and evaluating such projections of suitability and connectivity with respect to the uncertainty of climate change forecasts. Beyond utility for vector control for AAT and HAT in Kenya and Tanzania, the methods we develop can inform management of biological resources in a variety of contexts, from the control of unwanted species to the conservation of threatened and endangered biodiversity.

## CONFLICT OF INTEREST

The authors have no conflicts of interest to declare.

## Supporting information

Fig S1

Fig S2

Fig S3

Fig S4

Fig S5

Fig S6

Fig S7

Fig S8

Fig S9

Table S1

Table S2

Supplementary Material

Supplementary Material

## Data Availability

All data for this study including tsetse fly genotypes, tsetse fly trapping localities, and landscape/environmental parameters are available at the Dryad Digital Repository: https://doi.org/10.6078/D1B715
