# Supplementary material for: A machine learning approach to integrating genetic and ecological data in tsetse flies (Glossina pallidipes) for spatially explicit vector control planning
Source: Evol Appl. 2021 May 5;14(7):1762–77. doi: 10.1111/eva.13237 (PMC8288027; doi:10.1111/eva.13237)

Figure 1S. Characterization of seasons based on mean precipitation. Monthly precipitation at sampling sites, grouped by season.

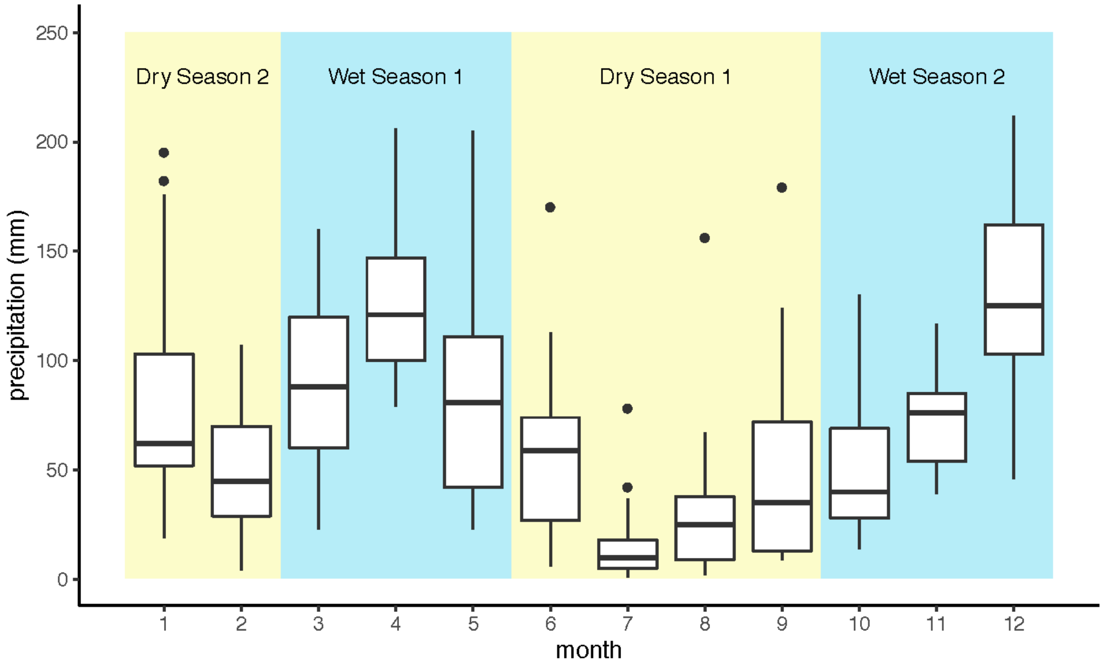

Supplement: Supplementary file 1 — Fig S1 [file EVA-14-1762-s006.pdf]
