# Supplementary material for: A machine learning approach to integrating genetic and ecological data in tsetse flies (Glossina pallidipes) for spatially explicit vector control planning
Source: Evol Appl. 2021 May 5;14(7):1762–77. doi: 10.1111/eva.13237 (PMC8288027; doi:10.1111/eva.13237)

Figure 2S. Genetic clustering results. Cluster membership assignments for each site based on a Discriminate Analysis of Principal Components (DAPC) results. Size of each box is proportional to the number of individuals assigned to that group (i.e. cluster).

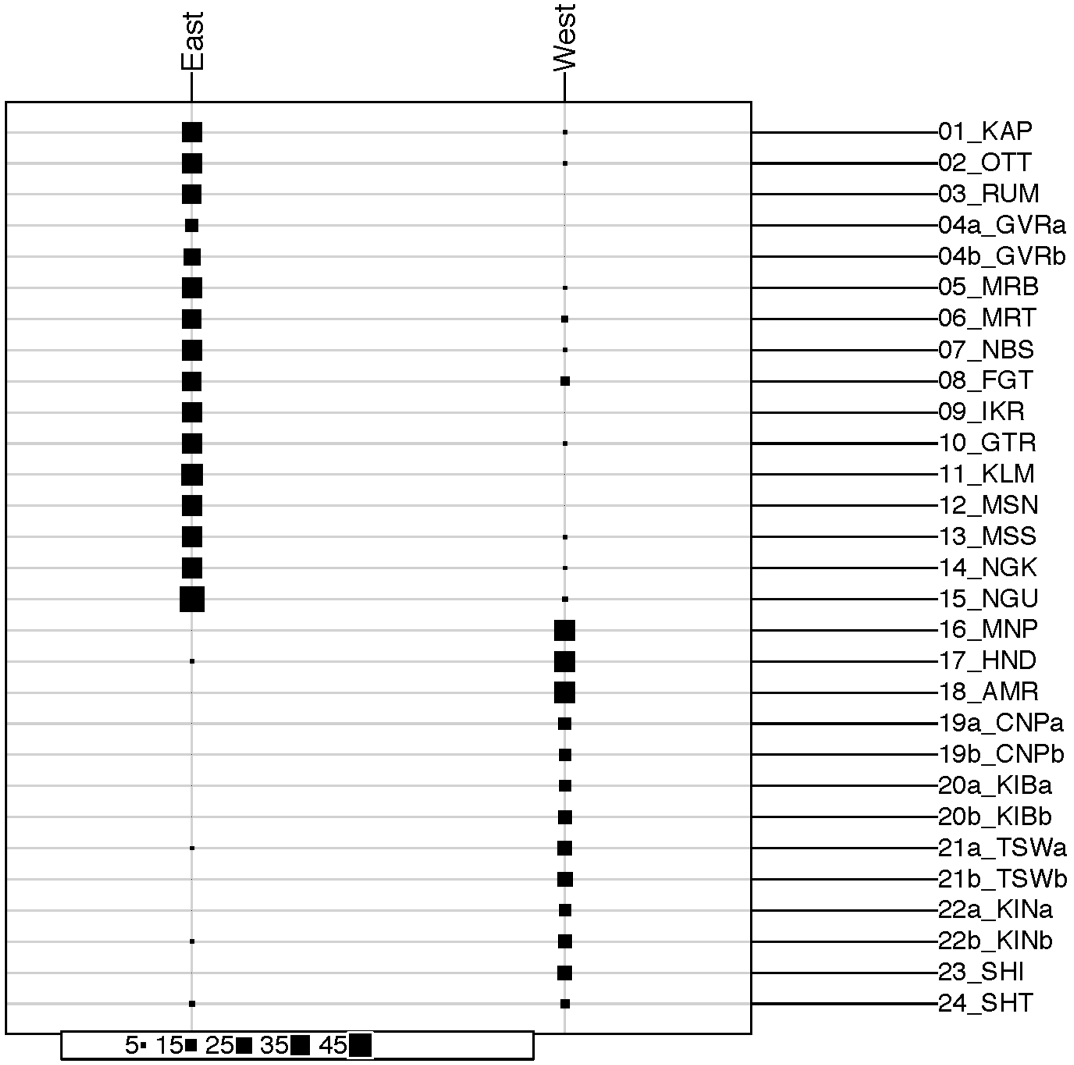

Supplement: Supplementary file 2 — Fig S2 [file EVA-14-1762-s009.pdf]
