# Supplementary material for: A machine learning approach to integrating genetic and ecological data in tsetse flies (Glossina pallidipes) for spatially explicit vector control planning
Source: Evol Appl. 2021 May 5;14(7):1762–77. doi: 10.1111/eva.13237 (PMC8288027; doi:10.1111/eva.13237)

Figure 3S. Spatial distributions of Cavalli-Sforza and Edwards' chord (CSE) genetic distance in the two major genetic clusters east and west of the Great Rift Valley. Density plots depict the distribution of CSE values for each genetic cluster. On the map, paths between sites within genetic clusters are colored according to their corresponding CSE value (darker values indicate higher genetic distance.

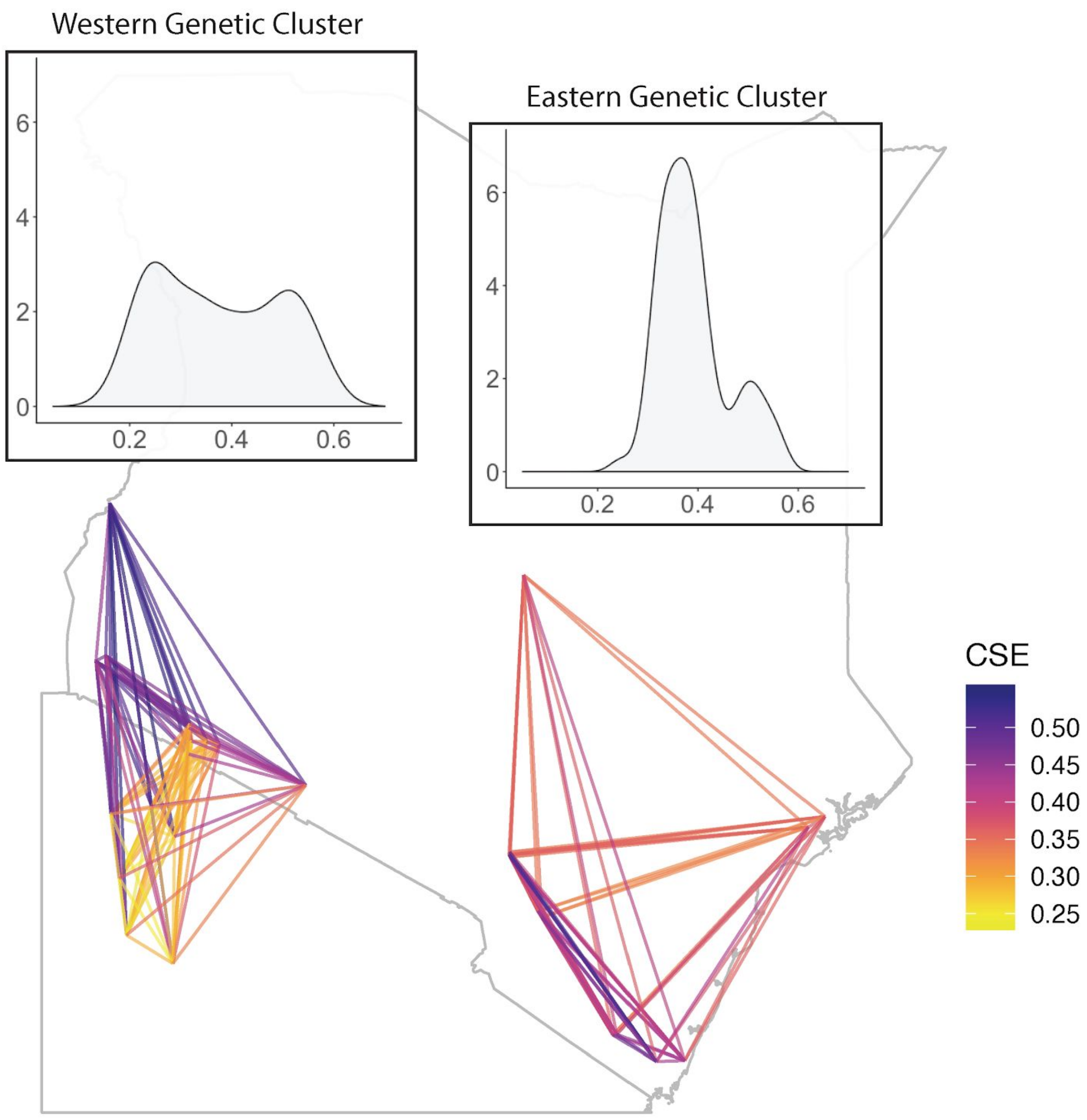

Supplement: Supplementary file 3 — Fig S3 [file EVA-14-1762-s011.pdf]
