# Supplementary material for: A machine learning approach to integrating genetic and ecological data in tsetse flies (Glossina pallidipes) for spatially explicit vector control planning
Source: Evol Appl. 2021 May 5;14(7):1762–77. doi: 10.1111/eva.13237 (PMC8288027; doi:10.1111/eva.13237)

Figure 4S. Mantel test for correlation of geographic and genetic distance. Results of the mantel tests for the **(A)** eastern and **(B)** western major genetic clusters, and **(C)** the western Serengeti sub-cluster (Bateta et al., 2020). The Lake Victoria sub-cluster (Bateta et al., 2020) was not included because of insufficient sample size. Plotted red lines are based on a linear model of Cavalli-Sforza and Edwards' chord (CSE) genetic distance and geographic distance (km). Simulated p-values are based on 999 replicates.

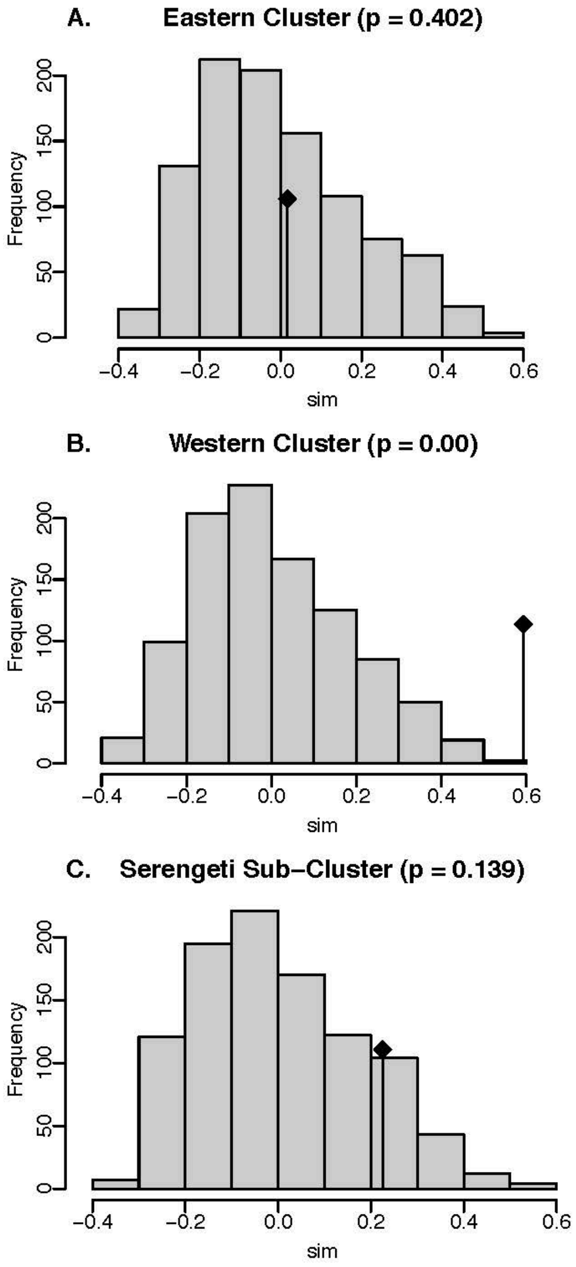

Supplement: Supplementary file 4 — Fig S4 [file EVA-14-1762-s013.pdf]
