# Supplementary material for: A machine learning approach to integrating genetic and ecological data in tsetse flies (Glossina pallidipes) for spatially explicit vector control planning
Source: Evol Appl. 2021 May 5;14(7):1762–77. doi: 10.1111/eva.13237 (PMC8288027; doi:10.1111/eva.13237)

Figure 5S. Projections of final models of habitat suitability and genetic connectivity. Raw projections of **(A)** the combined habitat suitability model and **(B)** the genetic connectivity model. The R-squared of the habitat suitability model (**A**) is based on the average R-squared of the 10 model replicates built using different sets of randomly sampled background points and all presence points. The R-squared of the genetic connectivity model (**B**) is the R-squared of the final model created with all of the data.

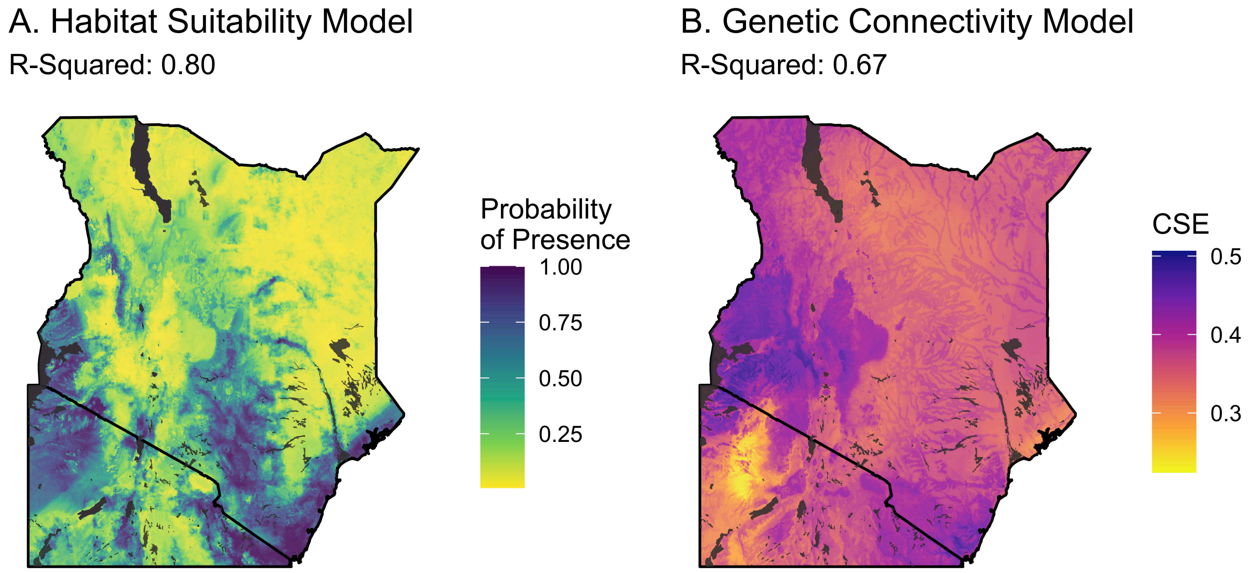

Supplement: Supplementary file 5 — Fig S5 [file EVA-14-1762-s005.pdf]
