# Supplementary material for: A machine learning approach to integrating genetic and ecological data in tsetse flies (Glossina pallidipes) for spatially explicit vector control planning
Source: Evol Appl. 2021 May 5;14(7):1762–77. doi: 10.1111/eva.13237 (PMC8288027; doi:10.1111/eva.13237)

Figure 6S. Variable importance for models of habitat suitability and genetic connectivity. Variable importance plots for **(A)** the 10 replicate habitat suitability models and **(B)** the final genetic connectivity model. The R package "randomForest" measures importance based on the increase in node purity (IncNodePurity), which is calculated by taking the decrease in the Residual Sum of Squares (RSS) as the result of splitting on each variable and averaging it across all trees (Liaw & Wiener, 2002). Variables correspond to those described in Table 1S. Abbreviations: Precipitation (Prec), Temperature (Temp).

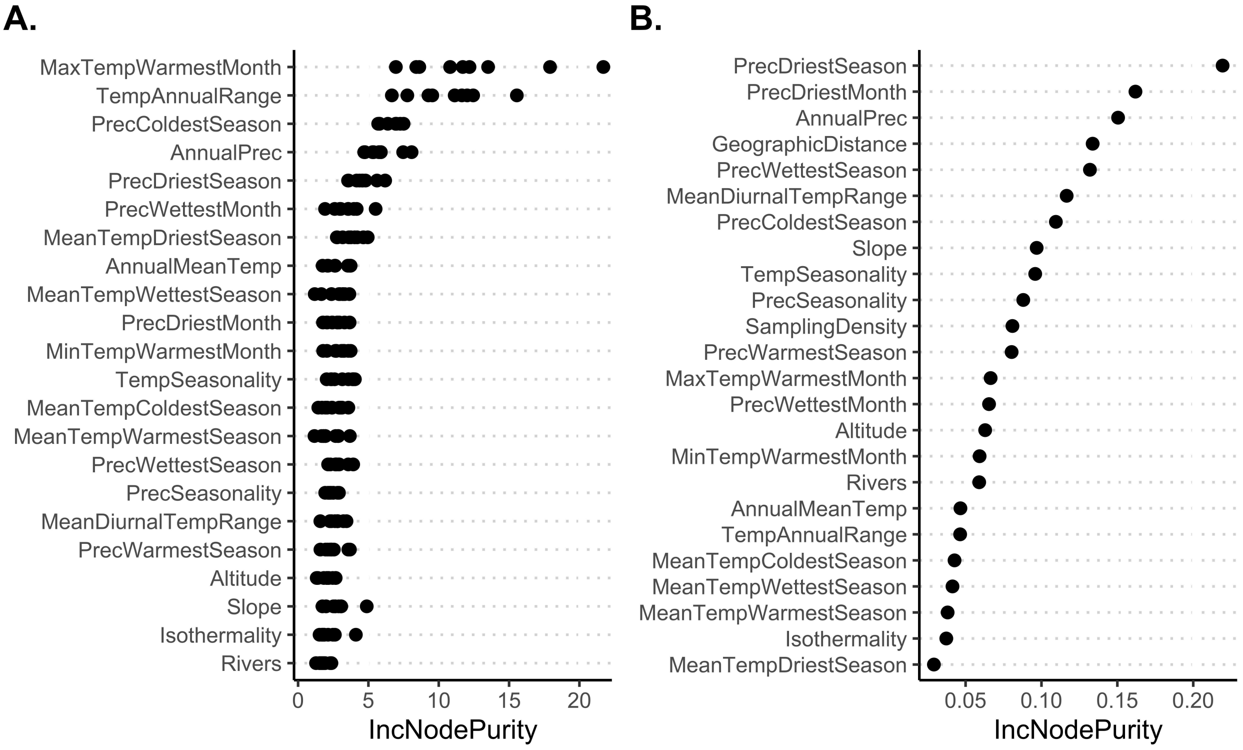

Supplement: Supplementary file 6 — Fig S6 [file EVA-14-1762-s001.pdf]
