# Supplementary material for: A machine learning approach to integrating genetic and ecological data in tsetse flies (Glossina pallidipes) for spatially explicit vector control planning
Source: Evol Appl. 2021 May 5;14(7):1762–77. doi: 10.1111/eva.13237 (PMC8288027; doi:10.1111/eva.13237)

Figure 7S. Comparison of observed and null RMSE distributions. Density plots of the observed distribution of RMSE values (red) from the connectivity model compared to 100 null distributions of RMSE values from models built using shuffled data (black). **(A)** Comparison of observed and null RMSE values from the model evaluation, **(B)** Comparison of the observed and null RMSE values from the spatial evaluation.

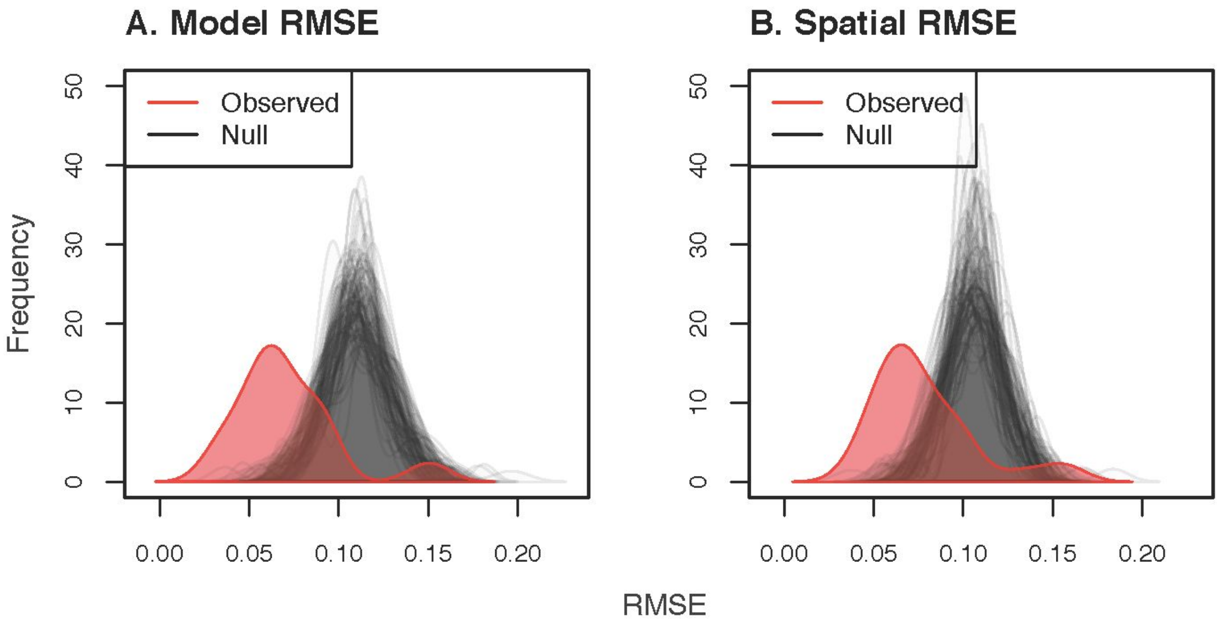

Supplement: Supplementary file 7 — Fig S7 [file EVA-14-1762-s003.pdf]
