# Supplementary material for: A machine learning approach to integrating genetic and ecological data in tsetse flies (Glossina pallidipes) for spatially explicit vector control planning
Source: Evol Appl. 2021 May 5;14(7):1762–77. doi: 10.1111/eva.13237 (PMC8288027; doi:10.1111/eva.13237)

Figure 8S. Distributions of observed and predicted genetic distances. Density plots depicting the distribution of Cavalli-Sforza and Edwards' chord (CSE) genetic distance values from the observed data (first plot) and from predictions of models with different variable combinations. The R squared values (RSQ) displayed are from random forest models created using the full dataset and the selected variables (as described in the plot titles). The p-values (p) in red are from Anderson-Darling k-sample tests used to compare the predicted distributions to the observed distribution (graphed in red). "Environmental" is abbreviated as "Env".

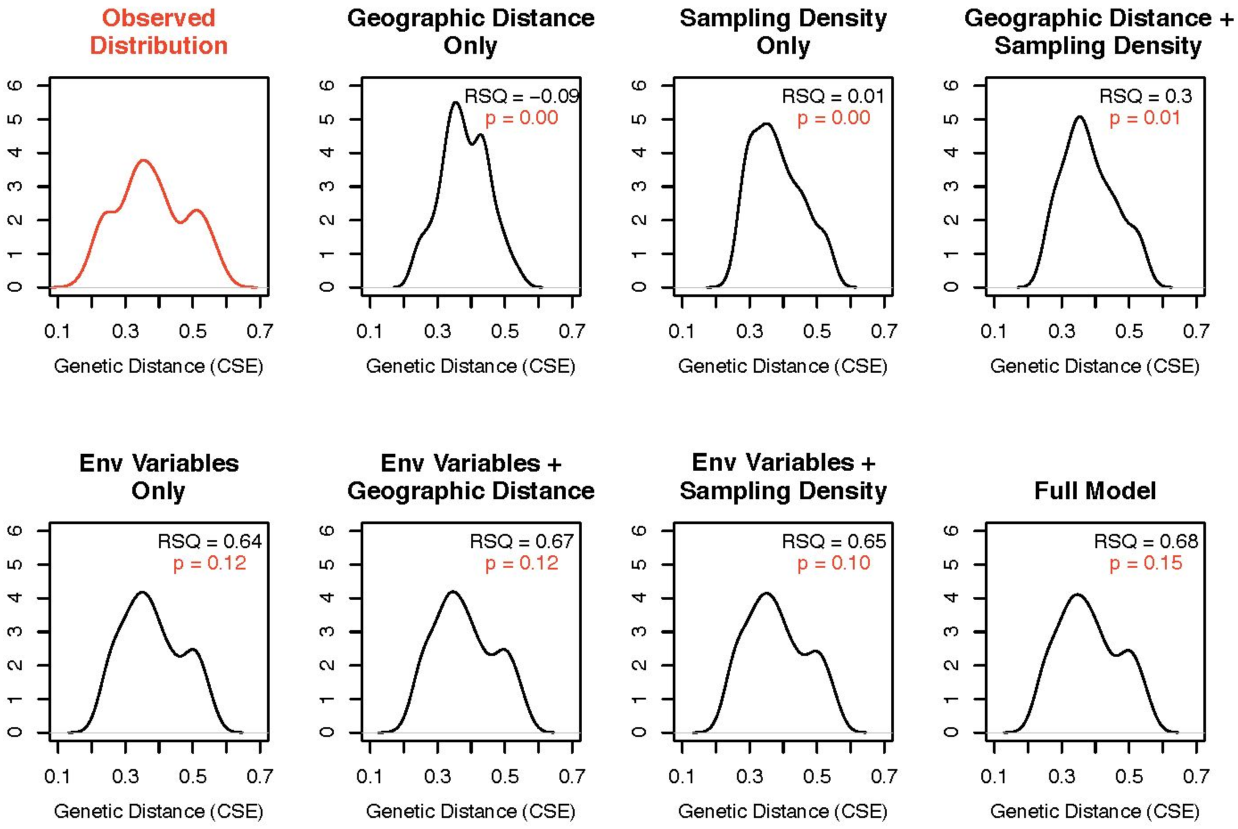

Supplement: Supplementary file 8 — Fig S8 [file EVA-14-1762-s004.pdf]
