# Supplementary material for: A machine learning approach to integrating genetic and ecological data in tsetse flies (Glossina pallidipes) for spatially explicit vector control planning
Source: Evol Appl. 2021 May 5;14(7):1762–77. doi: 10.1111/eva.13237 (PMC8288027; doi:10.1111/eva.13237)

Figure 9S. Comparison of random forest and simple linear regression model projections. Both models were built using the same response (CSE) and predictor variables. The left column of graphs are projections of the linear model. The right column of graphs are projections of the random forest model. The top row of graphs are maps set to the default scales (range of each projection). The bottom row of graphs are maps set to the scale of the observed data (range of observed CSE values).

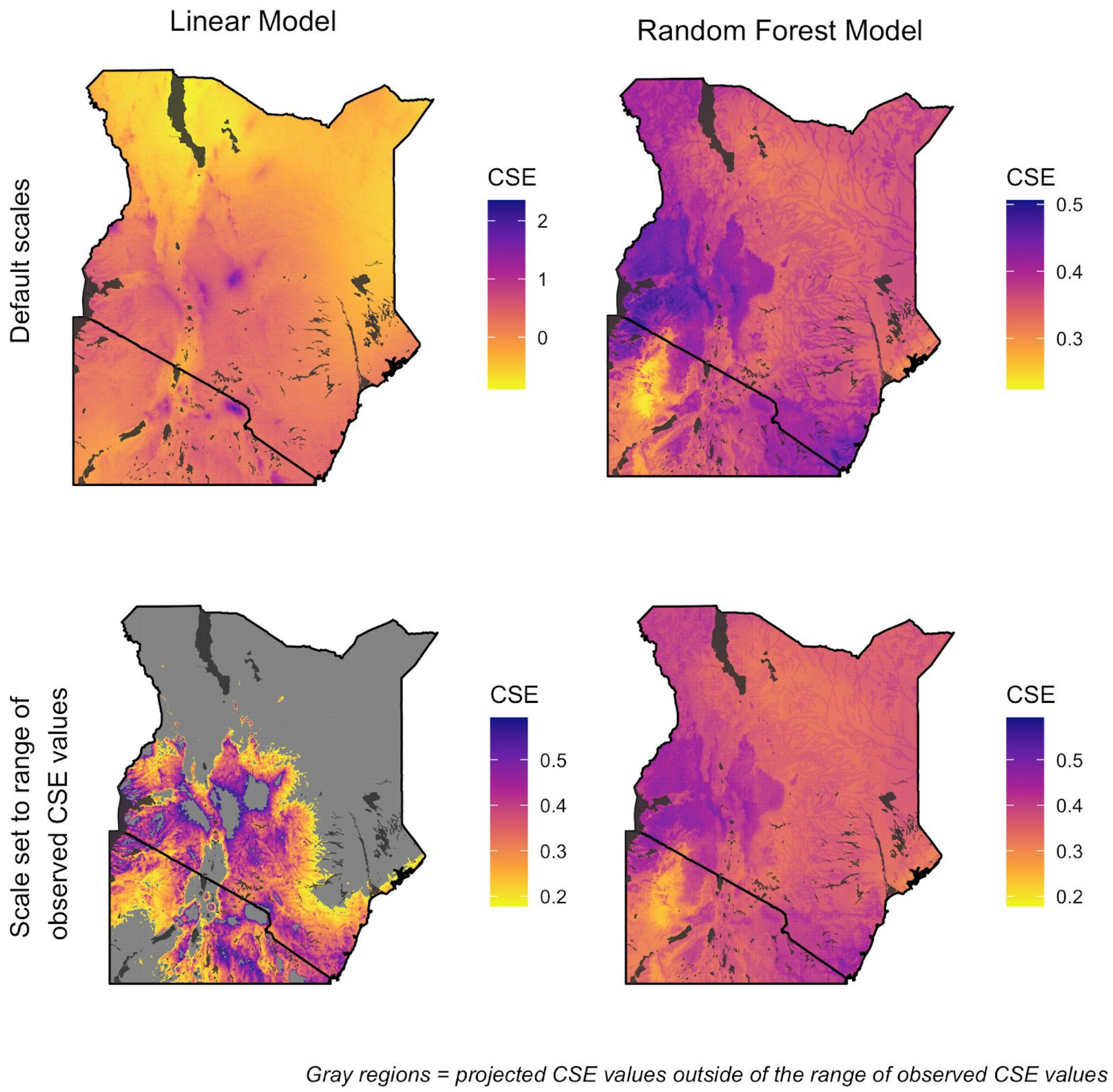

Supplement: Supplementary file 9 — Fig S9 [file EVA-14-1762-s010.pdf]
