# Supplementary material for: A machine learning approach to integrating genetic and ecological data in tsetse flies (Glossina pallidipes) for spatially explicit vector control planning
Source: Evol Appl. 2021 May 5;14(7):1762–77. doi: 10.1111/eva.13237 (PMC8288027; doi:10.1111/eva.13237)
Supplement: Supplementary file 10 — Table S1 [file EVA-14-1762-s008.pdf]

Table 1S: Environmental variables included as predictors in machine learning models. Complete list of 22 environmental variables. All "BIO" (Bioclimatic) variables were created using CHELSA data and the R package "biomod2" based on the bioclimatic variables from Worldclim (Thuiller et al., 2019; Karger et al., 2017). Bioclimatic variables ending in "S" are seasonal calculations of synonymous quarterly bioclimatic variables based on the precipitation cycles of Kenya (Figure 1S).

Variable	Description	Unit
BIO1	Annual mean temperature	K × 10
BIO2	Mean diurnal temperature range: mean of the monthly (max temp − min temp)	K × 10
BIO3	Isothermality (bio2/bio7 × 100)	K × 10
BIO4	Temperature seasonality: standard deviation × 100	K × 1000
BIO5	Max temperature of the warmest month	K × 10
BIO6	Min temperature of the warmest month	K × 10
BIO7	Temperature annual range	K × 10
BIO12	Annual precipitation	mm
BIO13	Precipitation of the wettest month	mm
BIO14	Precipitation of the driest month	mm
BIO15	Precipitation seasonality: coefficient of the variation	mm
BIO8S	Mean temperature of the wettest season	K × 10
BIO9S	Mean temperature of the driest season	K × 10
BIO10S	Mean temperature of the warmest season	K × 10
BIO11S	Mean temperature of the coldest season	K × 10
BIO16S	Precipitation of the wettest season	mm
BIO17S	Precipitation of the driest season	mm
BIO18S	Precipitation of the warmest season	mm
BIO19S	Precipitation of the coldest season	mm
Slope	Slope	dimensionless
Altitude	Altitude	m × 10
Rivers	Kernel density of surface waters	% river per m^2
