# Supplementary material for: A machine learning approach to integrating genetic and ecological data in tsetse flies (Glossina pallidipes) for spatially explicit vector control planning
Source: Evol Appl. 2021 May 5;14(7):1762–77. doi: 10.1111/eva.13237 (PMC8288027; doi:10.1111/eva.13237)
Supplement: Supplementary file 11 — Table S2 [file EVA-14-1762-s012.pdf]

Table 2S: Comparison of observed and predicted distributions of genetic distance. Table of results from Anderson Darling k-means tests comparing the (a) observed Cavalli-Sforza and Edwards' chord (CSE)genetic distance to predicted distributions based on models with environmental predictors of increasing complexity: (b) geographic distance only, (c) sampling density only, (d) geographic distance and sampling density, (e) environmental variables only, (f) environmental variables and geographic distance, (g) environmental variables and sampling density, and (h) the final full model with all environmental variables, geographic distance, and sampling density. Values in the lower triangle of this table are p-values and values in the upper triangle are Anderson-Darling Criterion values (with Anderson Darling standardized test statistics in parenthesis).

	(a)	(b)	(c)	(d)	(e)	(f)	(g)	(h)
(a) Observed Distribution	-	6.17 (6.83)	4.82 (5.04)	4.23 (4.27)	1.81 (1.06)	1.78 (1.03)	1.93 (1.23)	1.64 (0.84)
(b) Geographic Distance	0.00	-	1.50 (0.66)	1.24 (0.31)	3.31 (3.05)	3.27 (3.00)	3.26 (2.98)	3.32 (3.06)
(c) Sampling Density	0.00	0.18	-	0.46 (-0.72)	2.27 (1.67)	2.24 (1.64)	2.37 (1.81)	2.27 (1.68)
(d) Geographic Distance + sampling Density	0.01	0.25	0.79	-	1.34 (0.45)	1.37 (0.48)	1.31 (0.41)	1.43 (0.56)
(e) Environmental Variables Only	0.12	0.02	0.07	0.22	-	0.07 (-1.23)	0.07 (-1.23)	0.07 (-1.23)
(f) Environmental Variables + Geographic Distance	0.12	0.02	0.07	0.21	1.00	-	0.06 (-1.24)	0.07 (-1.23)
(g) Environmental Variables + Sampling Density	0.10	0.02	0.06	0.23	1.00	1.00	-	0.07 (-1.22)
(h) Full Model	0.15	0.02	0.06	0.19	1.00	1.00	1.00	-
