# Supplementary material for: A machine learning approach to integrating genetic and ecological data in tsetse flies (Glossina pallidipes) for spatially explicit vector control planning
Source: Evol Appl. 2021 May 5;14(7):1762–77. doi: 10.1111/eva.13237 (PMC8288027; doi:10.1111/eva.13237)
Supplement: Supplementary file 12 — Supplementary Material [file EVA-14-1762-s002.pdf]

SUPPLEMENTAL METHODS AND RESULTS

Table of Contents:

I. Habitat Suitability: Background Points.

II. Genetic Connectivity

  A. Population structure

  B. Accounting for spatial auto-correlation

  C. Model Evaluation

  D. Comparison of Random Forest and Linear Models

III. Creation of the Bivariate Map

I. Habitat Suitability: Background Points

We used 10 independent sets of 100 randomly sampled points across the geographic scope of our study to act as background points in our habitat suitability model. 100 randomly sampled points approximately matches the number of unique presence points at one kilometer resolution, which has been demonstrated to maximize accuracy in species distribution models (Barbet-Massin et al., 2012). We used each of the 10 sets of background points to build and evaluate 10 replicate habitat suitability models. For each of the 10 replicate models, we built a random forest suitability model using all 449 data points (349 presence points from our field-survey and a set of 100 background points). The 10 replicate models were projected across Kenya and part of Tanzania to create 10 maps of predicted habitat suitability, one for each set of background points. The mean of the 10 model projections was used to create our final habitat suitability model.

II. Genetic Connectivity

(A) Population Structure: We performed Discriminant Analysis of Principal Components (DAPC) using the R package "adegenet v2.0.1" (Jombart et al., 2008) to confirm the number of distinct genetic clusters in our data matched previous results (Okeyo et al., 2017, 2018; Bateta et al., 2020). The Discriminant Analysis of Principal Components (DAPC) results supported previous findings by Bateta et al. (2020), indicating two major genetic clusters to the east and west of the Great Rift Valley (Fig 2S, Fig 3S). Amongst the new samples, the majority of individuals from OTT clustered with the west (29 out of 30), whereas all individuals from CNP and AMR clustered with the east. Pairwise CSE genetic distances between sampling sites (no. of sampling sites = 29) ranged from 0.18 to 0.59 and had a mean of 0.38 (SD = 0.10). In the east (no. of sampling sites = 13), the CSE genetic distances ranged from 0.24 to 0.56 and had a mean of 0.39 (SD = 0.07). In the west (no. of sampling sites = 16), the CSE genetic distances ranged from 0.18 to 0.59 and had a mean of 0.37 (SD = 0.12). Density plots of genetic distance indicate that the east has predominantly intermediate genetic distance (with a few high genetic distances connected to a single sampling site, SHT) whereas the west has a combination of high and low genetic distance, north of and within the Serengeti ecosystem, respectively (Fig 3S).

(B) Accounting for Spatial Auto-correlation: Correlation of genetic distance and geographic distance was confirmed in some subsets of the current dataset with a Mantel test (Mantel, 1967) completed in the R package "ade4" (Bateta et al, 2020; Dray & Dufour, 2007). The results of the Mantel tests showed that geographic distance has a significant effect on genetic distance in the west, but not the east ($r = 0.57$ and -0.06, p-value = 0.003 and 0.567, respectively, Fig 4S), and not in the westerneastern sub-cluster with sufficient sampling, which was made up of sampling sites from the Serengeti ecosystem (Bateta et al., 2020; $r = 0.13$, p-value = 0.23, Fig 4S).

Based on an alpha level of 0.05, these results indicate that closer sampling sites have a smaller genetic distance than expected by chance in the west, but that there is no evidence for isolation by distance in the east or in the western Serengeti sub-cluster. This suggests that the significant relationship between geographic and genetic distance in the western cluster was due to genetic substructure and not true isolation by distance. Therefore, geographic distance alone is unlikely to be a strong predictor of genetic distance in this system.

To account for spatial auto-correlation as the result of uneven sampling and the correlation between genetic distance and geographic distance we included two additional explanatory variables, (i) sampling density (ii) and geographic distance in our genetic connectivity model: (i) Kernel density of sampling effort was estimated as the average number of sampling sites per 50 square meters within a moving window of 30 km using the R package "KernSmooth" (Shi et al., 2019; Souris et al., 2019), and saved at 1 km resolution. The median sampling density was then estimated as with the 22 environmental variables. (ii) The straight-path geographic distance in km was estimated by summing the number of pixels in each path. Although including both of these variables may be somewhat redundant because of their correlation ($r = -0.75$), the machine learning approach we use is robust to use of multiple correlated variables (Breiman, 2001; Liaw & Wiener, 2002), and including both of these variables was expected to improve our ability to account for spatial auto-correlation.

(C) Model Evaluation: To evaluate the connectivity model, we used leave-one-out cross-validation. For each of the 29 runs, we removed one of the 29 sampling sites and the associated pairwise paths for testing. This resulted in a testing set of 12 paths (6.06% of the total paths) for sampling sites from the eastern cluster, and 15 paths (7.58% of the total paths) for sampling sites from the western cluster. We then trained the model on the remaining 28 sampling sites (93.94%

and 92.42% of the total paths in the east and west, respectively). Root Mean Square Error (RMSE) of the testing sets were calculated for each run. By using this approach, we could evaluate the effectiveness of the model at predicting "new" sampling sites that had not been included in the training data. For each leave-one-out cross-validation run and for the final model, we projected the models to get maps of predicted genetic connectivity. We estimated the accuracy of the projections by extracting the median CSE genetic distance along straight paths from the testing data and comparing these values to the observed genetic distance.

In order to provide a benchmark for our RMSE values, we generated 100 null models trained on randomly shuffled data (i.e. data with no relationship between genetic distance and the predictor variables) for each of the 29 sites. We calculated the model RMSE and the spatial RMSE (i.e. RMSE based on the projections) for each of these null models to get distributions of 100 null values for each site. We compared each of the 100 sets of null RMSE values to the observed set of RMSE values using Welch's t-tests. We also compared the observed model and spatial RMSE values from our model to those of the null models (Fig 7S).

The observed set of RMSE values was significantly lower than each of the 100 sets of null values (mean = 0.11, SD = 0.02) in all 100 repetitions of this procedure ($t(45.91) = 6.47$, $p < 0.05$; t-statistic and degrees of freedom are averages of all 100 t-tests; Fig 7SA). The majority of our testing sites (23 out of 29 sites) had observed model RMSE values less than 90% of the null RMSE values. Only two sites (SHT and NGU; Fig 3B) had observed model RMSE values greater than 90% of the null distribution.

The observed set of spatial RMSE values was significantly lower than each of the 100 sets of null spatial RMSE values (mean = 0.11, SD = 0.02) in all 100 repetitions of this procedure ($t(42.75) = 4.51$, $p < 0.05$; t-statistic and degrees of freedom are averages of all 100 t-tests; Fig

7SB). The majority of our testing sites (21 out of 29) had observed spatial RMSE values less than 90% of the null spatial RMSE values. Only three sites (SHT, NGU, and KAP, Fig 3B) had observed spatial RMSE values greater than 90% of the null spatial RMSE distribution.

In order to determine whether the inclusion of environmental data in our model of connectivity resulted in an improvement from a model purely based on geographic distance or sampling density, we also compared the observed and predicted genetic distance distributions from models with different variable combinations using Anderson-Darling k-means tests with the R package "kSamples" (Scholz & Zhu, 2019). Results from these tests indicated that the observed distribution was significantly different from that predicted based on geographic distance alone ($p < 0.05$), but was not significantly different from that based on our full model ($p = 0.15$). Table 2S, Fig 8S). This suggests that inclusion of the environmental data was necessary to predict genetic connectivity.

(D) Comparison of Random Forest and Linear Models: To validate the selection of random forest regression for modelling connectivity, we compared our model performance to that of a simple linear model. The lm() function was used to build and evaluate a linear model using the same formula and leave-one-out cross-validation procedure used for our random forest model. The spatial RMSE for the linear model (mean = 0.12) was significantly greater than that of the random forest model (mean = 0.08; $t(46.16) = 3.74$, $p < 0.05$). Comparison of the projections from these two models illustrate how the non-linear, flexible nature of random forest regression allows for the capture of greater spatial heterogeneity in connectivity (Fig 9S). In addition, the range of projected CSE values from the linear model (min = -0.89, max = 2.35) extends far outside the range of observed CSE values from the input data (min = 0.18, max = 0.59) and the range of projected CSE values from the random forest model (min = 0.22, max = 0.50). The unrealistic

genetic distance values predicted by the linear regression suggest that the spatial projection of this model is unlikely to be an accurate map of connectivity (Fig 9S).

III. Creation of the Bivariate Map

To create the bivariate map both the habitat suitability model projection and the genetic connectivity model projection were scaled from zero to one. The scaling was necessary to make the units comparable and enable the calculation of 10% quantiles for the bivariate map. Because the projection of the genetic connectivity model was in units of distance, and we needed units of connectivity for the bivariate map, we transformed the scaled projection to $1 - scaled\ genetic\ distance$ to get a map for which increasing values represent increasing connectivity. The final bivariate color matrix consisted of breaks along the 10% quantiles for the habitat suitability model on one axis and the scaled and transformed genetic connectivity on the other axis. Code for the creation of the bivariate map is provided in File S2.
