# Supplementary material for: A machine learning approach to integrating genetic and ecological data in tsetse flies (Glossina pallidipes) for spatially explicit vector control planning
Source: Evol Appl. 2021 May 5;14(7):1762–77. doi: 10.1111/eva.13237 (PMC8288027; doi:10.1111/eva.13237)
Supplement: Supplementary file 13 — Supplementary Material [file EVA-14-1762-s007.pdf]

S2 Bivariate Map Code

A machine learning approach to integrating genetic and ecological data in tsetse flies (*Glossina pallidipes*) for spatially explicit vector control planning

Anusha Bishop, Giuseppe Amatulli, Chaz Hyseni, Evlyn Pless, Rosemary Bateta, Winnie A. Okeyo, Paul O. Mireji, Sylvance Okoth, Imna Malele, Grace Murilla, Serap Aksoy, Adalgisa Caccone, and Norah Saarman

Load libraries

```
#libraries for mapping
library(raster)
```

```
## Loading required package: sp
```

```
library(rgdal)
```

```
## rgdal: version: 1.5-16, (SVN revision 1050)
## Geospatial Data Abstraction Library extensions to R successfully loaded
## Loaded GDAL runtime: GDAL 3.1.1, released 2020/06/22
## Path to GDAL shared files: /Library/Frameworks/R.framework/Versions/4.0/Resources/lib
rary/rgdal/gdal
## GDAL binary built with GEOS: TRUE
## Loaded PROJ runtime: Rel. 6.3.1, February 10th, 2020, [PJ_VERSION: 631]
## Path to PROJ shared files: /Library/Frameworks/R.framework/Versions/4.0/Resources/lib
rary/rgdal/proj
## Linking to sp version:1.4-2
## To mute warnings of possible GDAL/OSR exportToProj4() degradation,
## use options("rgdal_show_exportToProj4_warnings"="none") before loading rgdal.
```

```
library(XML)
library(classInt)
library(rgeos)
```

```
## rgeos version: 0.5-5, (SVN revision 640)
##  GEOS runtime version: 3.8.1-CAPI-1.13.3
##  Linking to sp version: 1.4-2
##  Polygon checking: TRUE
```

```
#library for constructing relative paths to files
library(here)
```

```
## here() starts at /Users/Anusha/Documents/GpdKenya
```

Load maps for plots

Shape files sourced from DIVA-GIS (March 2020; http://www.diva-gis.org)

```
#define CRS and extent
crs.geo <- CRS("+proj=longlat +ellps=WGS84 +datum=WGS84 +no_defs")
ext <- extent(33.7, 42.5, -4.8, 5)

#SpatialPolygon Maps
KenyaMap <- readOGR(here("KEN_adm","KEN_adm0.shp"))
```

```
## OGR data source with driver: ESRI Shapefile
## Source: "/Users/Anusha/Documents/GpdKenya/KEN_adm/KEN_adm0.shp", layer: "KEN_adm0"
## with 1 features
## It has 70 fields
## Integer64 fields read as strings:  ID_0 OBJECTID_1
```

```
TzaMap <- readOGR(here("TZA_adm","TZA_adm0.shp"))
```

```
## OGR data source with driver: ESRI Shapefile
## Source: "/Users/Anusha/Documents/GpdKenya/TZA_adm/TZA_adm0.shp", layer: "TZA_adm0"
## with 1 features
## It has 70 fields
## Integer64 fields read as strings:  ID_0 OBJECTID_1
```

```
TzaKenMap <- bind(KenyaMap,TzaMap)
TzaKenMap <- crop(TzaKenMap,ext)

#water maps
kenya_water <- readOGR(here("KEN_wat","KEN_water_areas_dcw.shp"))
```

```
## OGR data source with driver: ESRI Shapefile
## Source: "/Users/Anusha/Documents/GpdKenya/KEN_wat/KEN_water_areas_dcw.shp", layer: "K
EN_water_areas_dcw"
## with 255 features
## It has 5 fields
```

```
tza_water <- readOGR(here("TZA_wat","TZA_water_areas_dcw.shp"))
```

```
## OGR data source with driver: ESRI Shapefile
## Source: "/Users/Anusha/Documents/GpdKenya/TZA_wat/TZA_water_areas_dcw.shp", layer: "T
ZA_water_areas_dcw"
## with 892 features
## It has 5 fields
```

```
TzaKenWater <- bind(kenya_water,tza_water)
TzaKenWater <- gBuffer(TzaKenWater, byid=TRUE, width=0)
```

```
## Warning in gBuffer(TzaKenWater, byid = TRUE, width = 0): Spatial object is not
## projected; GEOS expects planar coordinates
```

```
TzaKenWater <- crop(TzaKenWater,ext)
```

Load habitat suitability and genetic connectivity model projections

```
#rescale to 0 to 1 and mask any values with probability of presence <= 0.05
r <- raster(here("SDMreps","gpd_tzaken_FAOcombo_sdm100_10repmean_Nov3.tif")) #load in SD
M
r[r <= 0.10]<-NA
r.min = cellStats(r, "min")
r.max = cellStats(r, "max")
r.scaled <- ((r - r.min) / (r.max - r.min))
sdm <- r.scaled
proj4string(sdm) <- crs.geo
#mask
sdm <- mask(sdm,TzaKenMap)

#rescale to 0 to 1
r <- raster(here("LOPOCV","MEDIAN","EBKM_AllData.tif")) #load in genetic connectivity su
rface
r.min = cellStats(r, "min")
r.max = cellStats(r, "max")
r.scaled <- ((r - r.min) / (r.max - r.min))
con <- 1 - r.scaled #this step is done so that increasing values = increasing connectivi
ty (instead of increasing genetic distance)
proj4string(con) <- crs.geo
#mask
con<-mask(con,TzaKenMap)

plot(con, zlim=c(0,1))
```

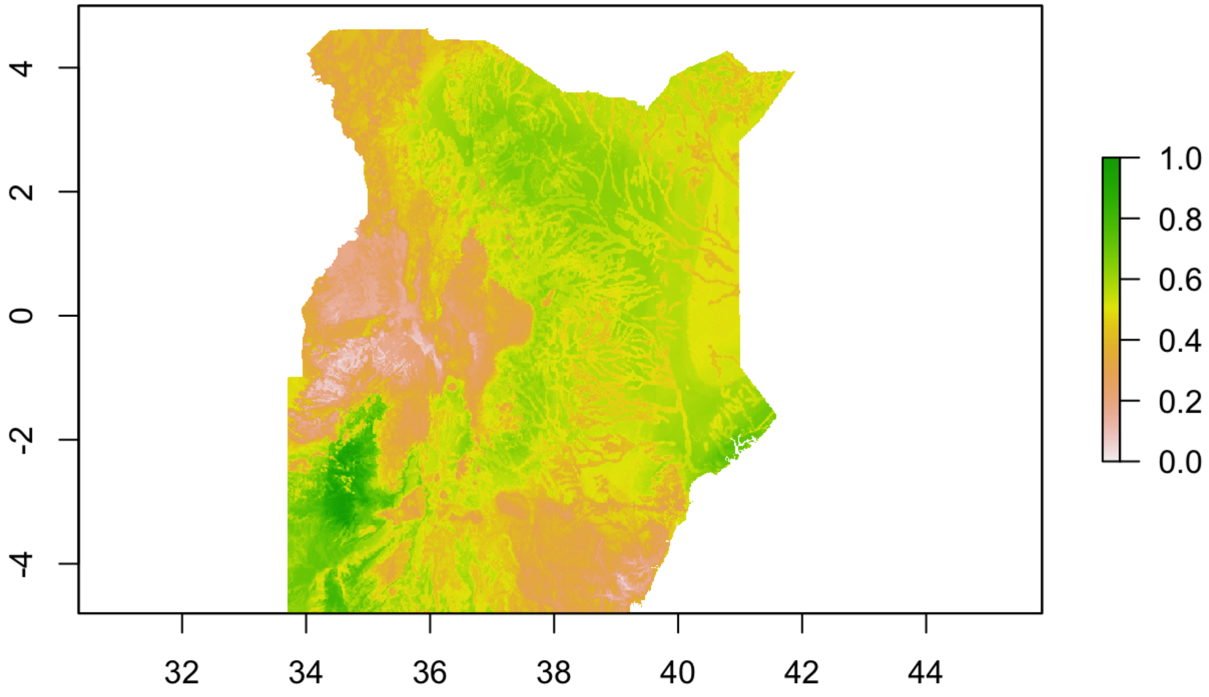


```
plot(sdm, zlim=c(0,1))
```

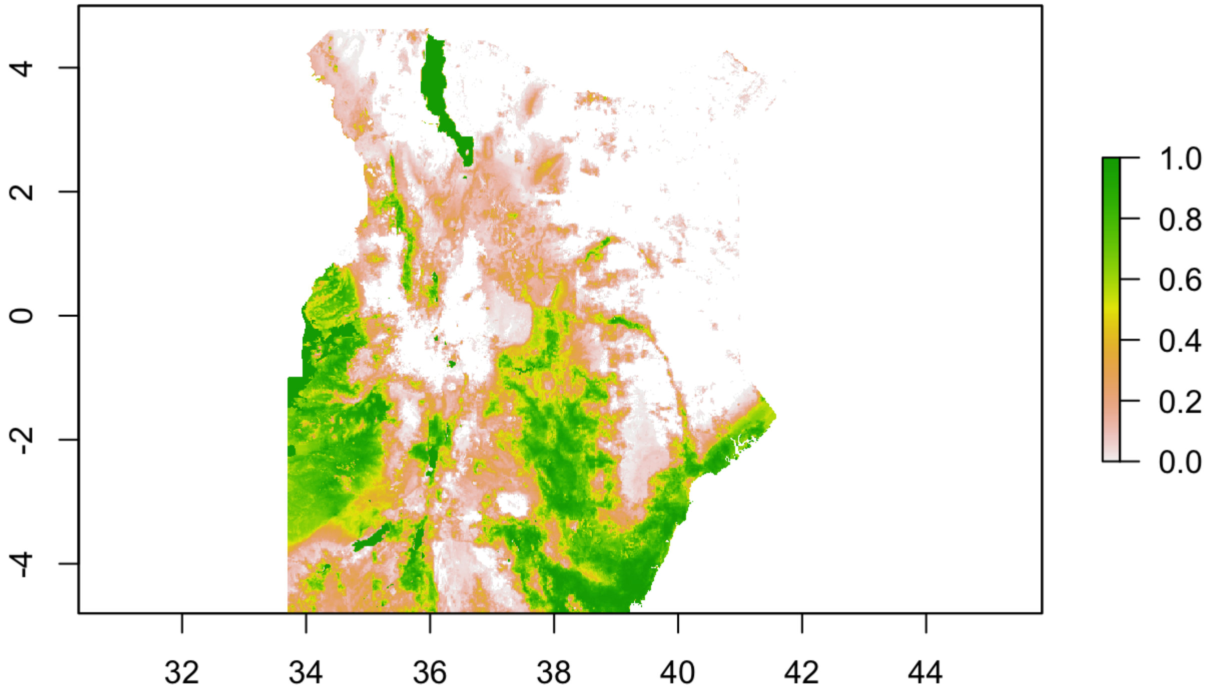

Code for Bivariate Map

Code sourced from: http://rfunctions.blogspot.com/2015/03/bivariate-maps-bivariatemap-function.html

```
###############################################
#create a color matrix for Bivariate map:
###############################################
colmat<-function(nquantiles=10, upperleft=rgb(0,150,235, maxColorValue=255), upperright=
"#700E40", bottomleft="grey", bottomright=rgb(255,230,15, maxColorValue=255), xlab="x la
bel", ylab="y label"){
  my.data<-seq(0,1,.01)
  my.class<-classIntervals(my.data,n=nquantiles,style="quantile")
  my.pal.1<-findColours(my.class,c(upperleft,bottomleft))
  my.pal.2<-findColours(my.class,c(upperright, bottomright))
  col.matrix<-matrix(nrow = 101, ncol = 101, NA)
  for(i in 1:101){
    my.col<-c(paste(my.pal.1[i]),paste(my.pal.2[i]))
    col.matrix[102-i,]<-findColours(my.class,my.col)}
  plot(c(1,1),pch=19,col=my.pal.1, cex=0.5,xlim=c(0,1),ylim=c(0,1),frame.plot=F, xlab=xl
ab, ylab=ylab, cex.lab=1.8)
  for(i in 1:101){
    col.temp<-col.matrix[i-1,]
    points(my.data,rep((i-1)/100,101),pch=15,col=col.temp, cex=1)}
  seqs<-seq(0,100,(100/nquantiles))
  seqs[1]<-1
  col.matrix<-col.matrix[c(seqs), c(seqs)]}

par(pty="s")
col.matrix<-colmat(nquantiles=10, xlab="Genetic Connectivity", ylab="Habitat Suitabilit
y")
```

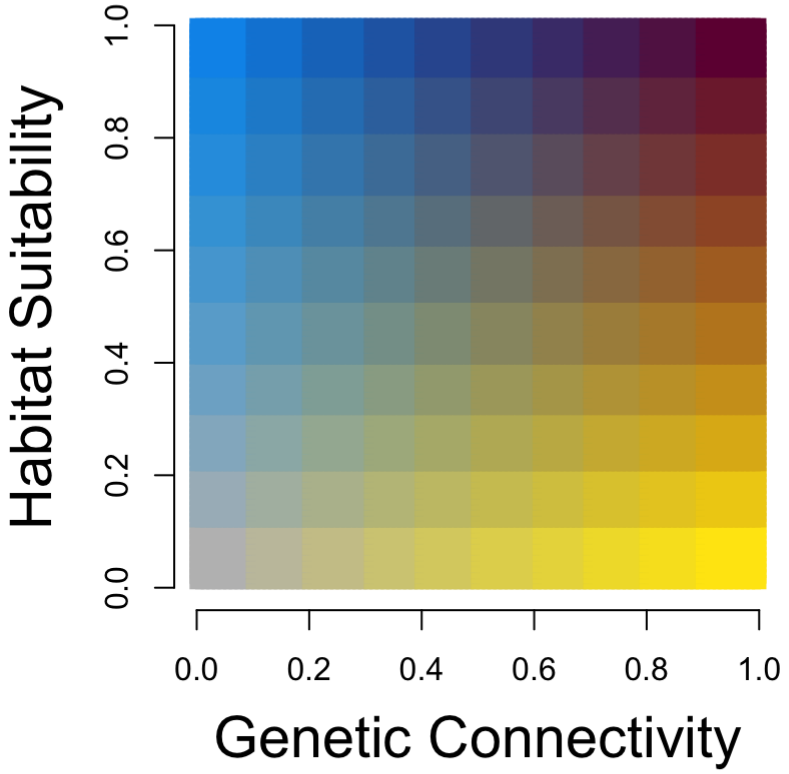

```
#############################################
#Build bivariate map function:
#############################################

rasterx <- con
rastery <- sdm
bivariate.map<-function(rasterx, rastery, colormatrix=col.matrix, nquantiles=10){
  quanmean<-getValues(rasterx)
  temp<-data.frame(quanmean, quantile=rep(NA, length(quanmean)))
  brks<-with(temp, quantile(temp,na.rm=T, probs = c(seq(0,1,1/nquantiles))))
  r1<-within(temp, quantile <- cut(quanmean, breaks = brks, labels = 2:length(brks),incl
ude.lowest = TRUE))
  quantr<-data.frame(r1[,2])
  quanvar<-getValues(rastery)
  temp<-data.frame(quanvar, quantile=rep(NA, length(quanvar)))
  brks<-with(temp, quantile(temp,na.rm=TRUE, probs = c(seq(0,1,1/nquantiles))))
  r2<-within(temp, quantile <- cut(quanvar, breaks = brks, labels = 2:length(brks),inclu
de.lowest = TRUE))
  quantr2<-data.frame(r2[,2])
  as.numeric.factor<-function(x) {as.numeric(levels(x))[x]}
  col.matrix2<-colormatrix
  cn<-unique(colormatrix)
  for(i in 1:length(col.matrix2)){
    ifelse(is.na(col.matrix2[i]),col.matrix2[i]<-1,col.matrix2[i]<-which(col.matrix2[i]=
=cn)[1])}
  cols<-numeric(length(quantr[,1]))
  for(i in 1:length(quantr[,1])){
    a<-as.numeric.factor(quantr[i,1]) #this gets what quantile the rasx value is in
    b<-as.numeric.factor(quantr2[i,1]) #this gets what quantile the rasy value is in
    cols[i]<-as.numeric(col.matrix2[b,a])} #this gets the corresponding col mat val for
 the two quants,
  r<-rasterx
  r[1:length(r)]<-cols
  return(r)}
```

Create and plot bivariate map

```
col.matrix <- colmat(nquantiles=10, xlab="genetic connectivity", ylab="habitat suitabili
ty")
```

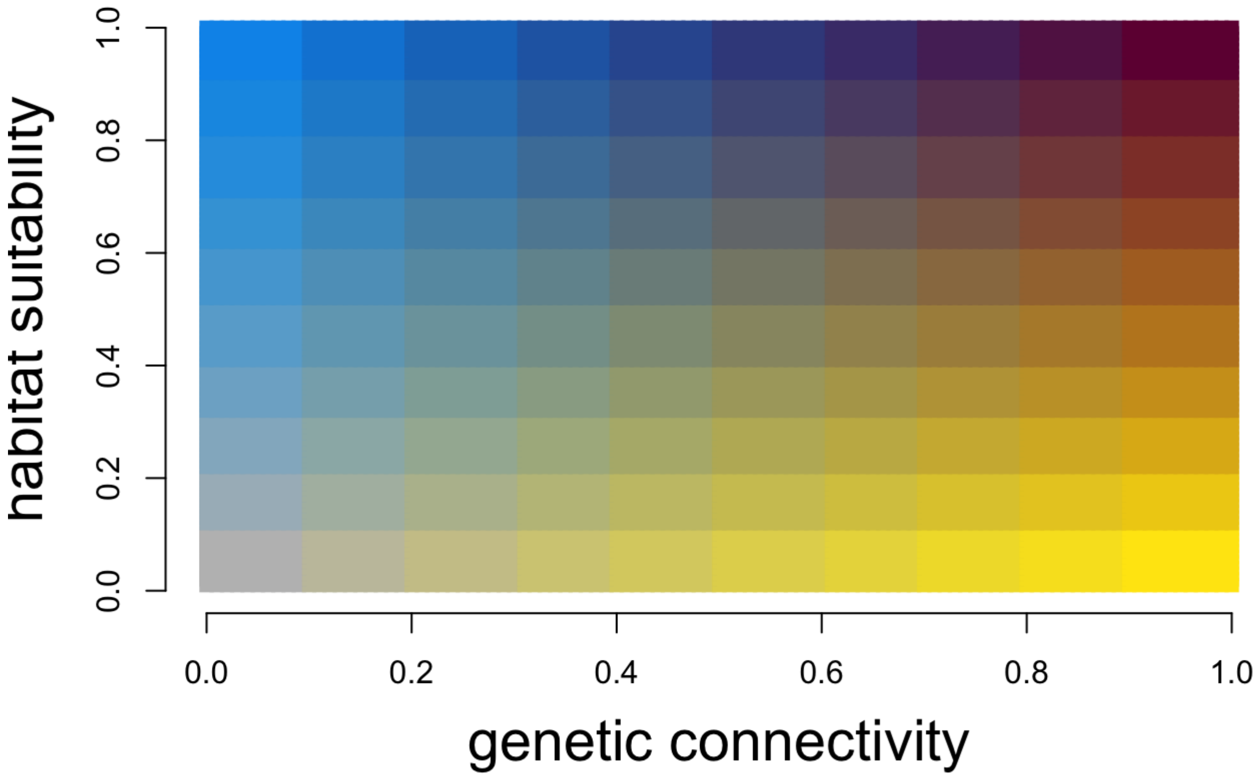


```
bivmap <- bivariate.map(con, sdm, colormatrix=col.matrix, nquantiles=10)
```

```
# Plot the bivariate map:
plot(bivmap,frame.plot=F,axes=F,box=F,add=F,legend=F,col=as.vector(col.matrix))
lines(TzaKenMap)
plot(TzaKenWater,add=T,col=rgb(0,0,0,alpha=0.70),legend=F)
```

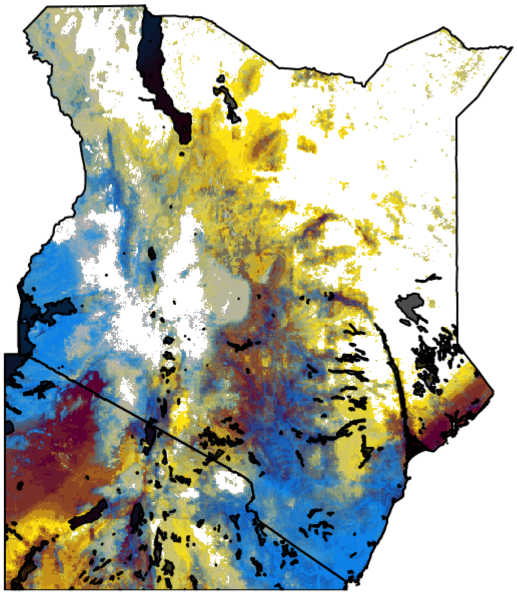

```
#bivariate legend
par(pty="s")
col.matrix<-colmat(nquantiles=10, xlab="Genetic Connectivity", ylab="Habitat Suitabilit
y")
```

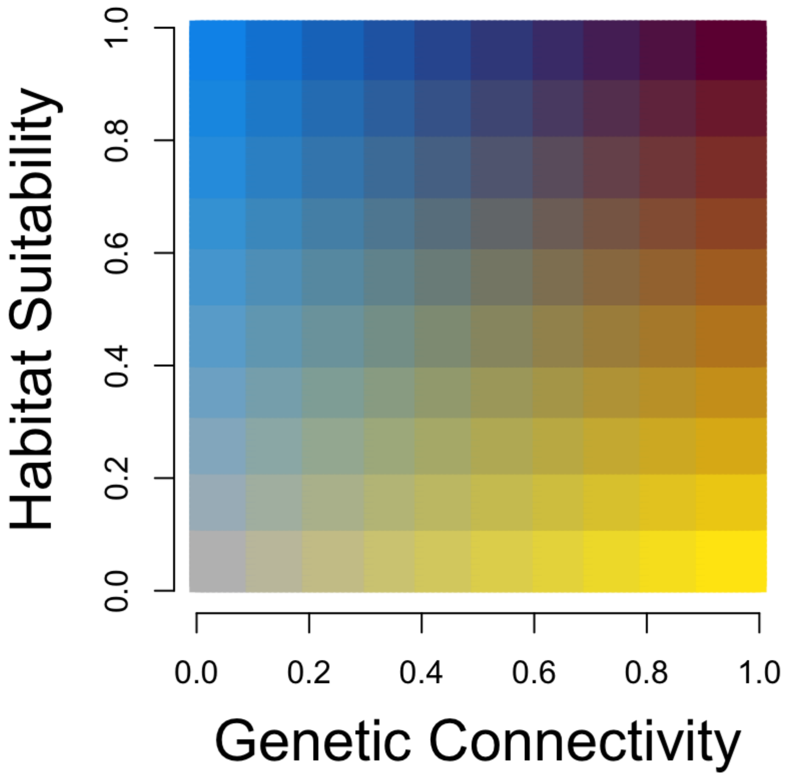
Habitat Suitability
0.0
0.2
0.4
0.6
0.8
1.0
0.0
0.2
0.4
0.6
0.8
1.0
Genetic Connectivity
